# RNA-Seq Analysis of IL-1B and IL-36 Responses in Epidermal Keratinocytes Identifies a Shared MyD88-Dependent Gene Signature

**DOI:** 10.3389/fimmu.2018.00080

**Published:** 2018-01-29

**Authors:** William R. Swindell, Maria A. Beamer, Mrinal K. Sarkar, Shannon Loftus, Joseph Fullmer, Xianying Xing, Nicole L. Ward, Lam C. Tsoi, Michelle J. Kahlenberg, Yun Liang, Johann E. Gudjonsson

**Affiliations:** ^1^Heritage College of Osteopathic Medicine, Ohio University, Athens, OH, United States; ^2^Department of Dermatology, University of Michigan, Ann Arbor, MI, United States; ^3^Department of Dermatology, Case Western Reserve University, Cleveland, OH, United States; ^4^Department of Internal Medicine, Division of Rheumatology, University of Michigan, Ann Arbor, MI, United States

**Keywords:** epidermis, ETS1, IL-1, inflammatory bowel disease, keratinocytes, NF-kappaB, RNA-seq

## Abstract

IL-36 cytokines have recently emerged as mediators of inflammation in autoimmune conditions including psoriasis vulgaris (PsV) and generalized pustular psoriasis (GPP). This study used RNA-seq to profile the transcriptome of primary epidermal keratinocytes (KCs) treated with IL-1B, IL-36A, IL-36B, or IL-36G. We identified some early IL-1B-specific responses (8 h posttreatment), but nearly all late IL-1B responses were replicated by IL-36 cytokines (24 h posttreatment). Type I and II interferon genes exhibited time-dependent response patterns, with early induction (8 h) followed by no response or repression (24 h). Altogether, we identified 225 differentially expressed genes (DEGs) with shared responses to all 4 cytokines at both time points (8 and 24 h). These involved upregulation of ligands (*IL1A, IL1B*, and *IL36G*) and activating proteases (*CTSS*) but also upregulation of inhibitors such as *IL1RN* and *IL36RN*. Shared IL-1B/IL-36 DEGs overlapped significantly with genes altered in PsV and GPP skin lesions, as well as genes near GWAS loci linked to autoimmune and autoinflammatory diseases (e.g., PsV, psoriatic arthritis, inflammatory bowel disease, and primary biliary cholangitis). Inactivation of MyD88 adapter protein using CRISPR/Cas9 completely abolished expression responses of such DEGs to IL-1B and IL-36G stimulation. These results provide a global view of IL-1B and IL-36 expression responses in epidermal KCs with fine-scale characterization of time-dependent and cytokine-specific response patterns. Our findings support an important role for IL-1B and IL-36 in autoimmune or autoinflammatory conditions and show that MyD88 adaptor protein mediates shared IL-1B/IL-36 responses.

## Introduction

The IL-36 cytokines (IL-36A, IL-36B, and IL-36G) are recent additions to the IL-1 family with potent pro-inflammatory effects on epithelial tissues in skin and other organs (1). These cytokines are produced by diverse immune cells, including T cells, dendritic cells, plasma cells, and monocytes, although in skin epidermal keratinocytes (KCs) appear to be the most prominent source (2). The role of IL-36 in skin pathology was first suggested by transgenic mouse models with IL-36A overexpressed in basal KCs, which yielded epidermal thickening and a pro-inflammatory phenotype exacerbated by deletion of the IL-36 receptor antagonist IL-36RN (3). Deletion of IL-36 receptor in mice was additionally protective against full development of imiquimod-induced dermatitis (4), further suggesting that IL-36 cytokines mediate inflammation secondary to disruption of epidermal homeostasis. More recently, evidence has emerged to suggest that IL-36 has an important role in diverse autoimmune conditions, including rheumatoid arthritis, systemic lupus erythematosus, and inflammatory bowel disease (IBD) (5–8). Within the intestine, for example, activation of IL-36 signaling appears to facilitate mucosal healing (5), and consistent with this *IL36A* and *IL36G* mRNAs are elevated in mucosa of ulcerative colitis patients (6). These findings have intensified research focus on IL-36 ligands and their regulatory proteins, with the hope that anti-IL-36 therapies can provide treatment options for cutaneous and non-cutaneous diseases, similar to the success of other anti-cytokine biologics demonstrating efficacy in recent years (9).

The emerging evidence implicating IL-36 in cutaneous disease has supported a role in psoriasis vulgaris (PsV) as well as a severe form of PsV known as generalized pustular psoriasis (GPP) (10). Mutations promoting IL-36A activation have been linked to GPP, including *IL36RN* missense mutations (11, 12) and inactivating mutations of the AP1S3 adaptor protein complex 1 (AP-1) subunit (13). By contrast, genome-wide association studies of PsV have thus far not uncovered disease-associated variants near IL-36A, IL-36B, IL-36G, or the antagonist IL-36RN, suggesting that genetic factors predisposing individuals toward PsV do not directly promote IL-36 hyperactivity. This may reflect differences in the immunological basis of PsV compared with GPP, with PsV and GPP mediated primarily by adaptive vs. innate/autoinflammatory responses, respectively (14). Nonetheless, PsV and GPP can co-occur in the same patient and possess shared pathognomonic features, such as neutrophil infiltration leading to development of pustules (GPP) or Munro’s microabscesses (PsV). With regard to the more common PsV, IL-36 expression is abundant in skin lesions and appears to at least provide a psoriatic biomarker, with higher expression in PsV lesions compared with lesions from other inflammatory skin conditions (e.g., eczema) (15). It is notable that in a large scale meta-analysis of skin samples from 237 psoriasis patients, the gene encoding IL-36G (*IL36G*) was 1 of only 5 genes with elevated expression in lesional skin from all 237 patients, indicating that elevated *IL36G* expression is a near-universal feature of PsV lesions (16). It was also recently shown that IL-36A is elevated in synovial tissues of patients with psoriatic and rheumatoid arthritis (compared with osteoarthritis), suggesting a possible role for IL-36 in the development of psoriatic arthritis (PsA) as well (17).

In skin, KCs are both a source of IL-36 and important cellular target that can be activated to undergo proliferation and release additional cytokines and chemokines (18). Within the epidermis, key extracellular events preceding IL-36 receptor stimulation have been well studied, although downstream signaling has been mostly studied in transformed cell lines and not primary skin cells (19). IL-36 cytokines are expressed as inactive precursors but are activated by KC- or neutrophil-derived proteases cathepsin S, cathepsin G, elastase, and proteinase-3 (4, 20). The IL-36 receptor is a heterodimer that includes IL-1Rrp2 (IL-36R) and IL-1R accessory protein (IL-1RAcP) subunits, with extracellular immunoglobulin and intracellular toll/IL-1 (TIR) domains shared with other receptors from the IL-1 cytokine family (1). The IL-1RAcP component of the IL-36 receptor is identical to that of the IL-1 receptor, which may contribute to overlapping effects of IL-1 and IL-36. Stimulation of the IL-36 receptor is expected to promote recruitment of the MyD88 adaptor protein to the TIR domain and activate the JNK, MAPK, and ERK1/2 pathways (19). This triggers a downstream expression response coordinated by key transcription factors, which in various cell types appear to include NF-kappaB and AP-1 (6, 19, 21, 22). It is unclear if these factors are activated by IL-36 in epidermal KCs, although it was shown that IL-36 cytokine expression is positively correlated with NF-kappaB abundance in PsV skin lesions (23).

This study uses RNA-seq to evaluate the gene expression response of primary epidermal KCs to stimulation by cytokines from the IL-1 family (IL-1B, IL-36A, IL-36B, and IL-36G). These ligands exhibit strong amino acid homology and interact with receptors sharing conserved domains and an identical IL-1RAcP subunit (24). The four cytokines were therefore included in this study to characterize potential differences and similarities, and we additionally replicate experiments at two time points (8 and 24 h) to identify early and late responses. We identify IL-1B- and IL-36-responsive genes, characterize known functions of such genes, and investigate relationships to psoriasis (PsV and GPP) based upon skin lesion transcriptome signatures. We further integrate these findings with those from GWA studies to assess whether IL-1B and IL-36 target genes are genetically associated with psoriasis or other autoimmune conditions. Finally, we assess the functional dependence of such genes on MyD88 adaptor protein activity and downstream transcription factors.

## Materials and Methods

### Normal Human Epidermal KC Culture

Normal human epidermal KC cultures were established from sun-protected skin of three unrelated donors (25). Skin biopsies were obtained from volunteer patients following protocols approved by the University of Michigan institutional review board (Ann Arbor, MI, USA, IRB No. HUM00037994). Cultures were maintained until 4 days post-confluence with serum-free medium (Medium 154, Invitrogen/Cascade Biologics, Portland, OR, USA). Only second or third passage cells were used in experiments with a calcium concentration of 0.1 mM. Cultures were starved of growth factors for a 24 h period before treatment with truncated recombinant human IL-1B, IL-36A, IL-36B, or IL-36G (10 ng/ml for IL-1B and for each of the IL-36 family cytokines—2,000 ng/ml; R&D Systems, Minneapolis, MN, USA). Experiments performed using IL-1B were carried out separately from those performed with IL-36 and paired with separate control (CTL) samples (8 h: two CTL and two IL-1B samples; 24 h: three CTL and three IL-1B samples). IL-36 experiment samples were processed independently and also included 8 and 24 h treatments (CTL, IL-36A, IL-36B, and IL-36 treatments; *n* = 3 per time point).

### High-Throughput cDNA Sequencing

The Illumina TruSeq mRNA Sample Prep v2 kit was used for high-throughput sequencing of cDNA (catalog no. RS-122-2001 and RS-122-2002) with approximately 0.1–3.0 μg of total RNA per sample used to generate mRNA by polyA purification. Reverse transcriptase and random primers were used to convert mRNA to cDNA following fragmentation, with final cDNA libraries purified and enriched using PCR (Kapa’s library quantification kit for Illumina Sequencing platforms; catalog no. KK4835; Kapa Biosystems, Wilmington, MA, USA). The Agilent TapeStation was used for cDNA quantification and quality assessment. The Illumina clonal amplification system (cBot) was used to cluster samples with adaptor barcodes to permit sequencing of 6 samples in each Illumina HiSeq 2000 flow cell lane (50-cycle single end).

### RNA-Seq Read Mapping and Quality CTL

Fastq files containing reads were filtered using Cutadapt to remove Illumina adaptor sequences and low quality reads, with a Phred33 quality score cutoff of 30 (-q option) and minimum read length of 20 bp (-minimum-length option) (26). The fastx-toolkit window based quality filter was also applied to remove reads with quality score less than 30 for 50% or more of the read length (27). Pre- and post-filter quality CTL statistics for each sample were calculated with FastQC (28). Reads that did not map to ribosomal sequences were obtained by initially running tophat2 (29) on reads for each sample, with default parameters and GTF file specifying ribosomal read locations only (UCSC hg19/hg38). Unmapped reads from this initial tophat2 run (i.e., reads not aligning to ribosomal RNA sequences) were then used for a second tophat2 run in which reads were mapped using a full GTF file specifying the locations of all human genes (UCSC hg19/hg38). Samtools was used to sort bam files and calculate alignment statistics (30). HTseq was used to tabulate the number of reads mapping to each known human gene, with only reads mapping unambiguously to a given gene included in the tabulation (option -m intersection-strict) (31). Cufflinks was used to calculate normalized FPKM and FPKM confidence intervals for quantification of gene expression (32). Mapping rates and expression profiling efficiency were calculated using the RSeQC and RNA-SeQC software packages (33, 34). Raw and processed sequencing data are available from Gene Expression Omnibus under the accession GSE109182.

### Dimensionality Reduction and Global Visualization of Cytokine Responses

Global visualization of genome-wide cytokine responses was facilitated by several techniques, including hierarchical cluster analysis, principal component (PC) response vectors (35), self-organizing maps (SOMs) (36), and Chernoff faces (37). SOMs were generated using the R Package “kohonen” (function: somgrid), and Chernoff faces were generated *via* the R package “aplpack” (function: faces). In the current context, Chernoff faces provide an easily interpreted and intuitive multivariate visualization complementing other methods of dimensionality reduction (37). Chernoff face components were scaled in proportion to cytokine FC estimates (cytokine/CTL) for 15 selected genes (face height: *QDPR*; face width: *ZNF316*; face structure: *MAT2B*; mouth height: *DECR1*; mouth width: *NEDD1*; smiling: *SMARCE1*; eye height *COPG2*; eye width: *METTL15*; hair height: *BCR*; hair width: *HDDC2*; hair style: *TBKBP1*; nose height: *TAB2*; nose width: *FSTL3*; ear width: *C2orf68*; and ear height: *NDOR1*). Likewise, colors of face components reflect FC estimates for the 15 genes (face: *QDPR, ZNF316*; lips: *QDPR, ZNF316, MAT2B*; eyes: *COPG2, METTL15*; hair: *BCR, HDDC2, TBKBP1*; nose: *TAB2, FSTL3*; and ears: *C2orf68, NDOR1*). The 15 genes were each chosen to represent a gene cluster identified by clustering all KC-expressed genes, with each cluster exhibiting a different response pattern among the 4 cytokines and both time points. The gene chosen to represent a given cluster was the gene with lowest average Euclidean distance between its response profile and all other genes in the cluster.

### Identification of Differentially Expressed Genes (DEGs)

Differentially expressed genes were identified from comparisons between cytokine-treated and corresponding CTL cells at each time point (8 and 24 h). Tests for differential expression were performed using protein-coding genes with detectable expression in at least 25% of samples involved in a given comparison. A gene was considered to have detectable expression in a given sample if the count per million mapped reads (cpm) was greater than 0.25 and if the lower limit on the FPKM 95% confidence interval was greater than 0. To evaluate differential expression, raw counts tabulated for each gene were first normalized using the weighted trimmed mean of *M*-values method (R package: edgeR and function: calcNormFactors) (38). Tests for differential expression were performed by fitting negative binomial generalized linear models (R package: edgeR and function: glmFit) (39), with negative binomial model dispersions estimated using the Cox–Reid-adjusted likelihood method (R package: edgeR and function: estimateGLMTrendedDisp) (40). Negative binomial models were generated using treatment (cytokine-stimulated vs. CTL cells) and cell line (line A, B, and C) as covariates. Differential expression *P*-values were obtained by comparing the full model (treatment + cell line) and reduced model (cell line only) within the likelihood ratio test framework (R package: edgeR; function: glmLRT) (39). Raw *P*-values from differential expression analyses were adjusted to CTL the false discovery rate (FDR) using the Benjamini–Hochberg method (41).

### Functional Analysis of DEGs

Differentially expressed genes were analyzed to assess enrichment for KEGG, GO BP, CC, and MF terms using the conditional hypergeometric test implemented in the GOstats R package (function: hyperGTest) (42). Annotated KEGG pathway diagrams were drawn using the pathview R package (43). Enrichment of medical subject heading (MeSH) terms was evaluated using the hypergeometric test implemented in the meshR R package (function: meshHyperGTest) (44). Likewise, enrichment of Disease Ontology categories was evaluated using the DOSE R package (function: enrichDO) (45). Comparisons with ranked gene lists generated from microarray data comparisons involving cytokine-treated KCs or human skin diseases were performed using a database assembled and described in a previous publication (46). The NHGRI-EBI GWAS catalog (release 2017-07-10) was used to determine if DEGs were enriched with respect to genes associated with particular human traits or diseases (hypergeometric test) (47). For analysis of genes near loci identified by GWA studies of PsV or PsA, we used the list of loci generated from a recent GWAS meta-analysis of these disease phenotypes (48).

### Transcription Factor Binding Site Analyses

DNA sequence regions 5,000 bp upstream of DEGs were evaluated for increased frequency of binding sites associated with human transcription factors or unconventional DNA-binding proteins (uDBPs). This was done by screening a set of 2,935 motifs compiled from multiple sources as described in a previous study (16). Generalized additive logistic regression models were used to evaluate the significance of motif count differences between DEGs and all other KC-expressed genes (49). Human transcription factor superfamily and family annotations were obtained from the TFClass database (50).

### Formalin-Fixed Paraffin-Embedded (FFPE) Tissue Analysis of PsV and GPP Skin Lesions

Gene expression in PsV and GPP lesions was compared using microarray samples generated from FFPE tissue samples as described in a previous study (10). These data had been generated from the Affymetrix Human Gene 2.1 ST array platform and are available through the Gene Expression Omnibus accession GSE79704. Microarray data files were normalized using robust multichip average (51), and the linear model procedures implemented in the R limma package were used to test for differential expression (52).

### N/TERTs Cell Culture

N/TERTs (53), an immortalized KCs line, was used with the kind permission of Dr. James G. Rheinwald for generation of knockout (KO) cell lines using non-homologous end joining *via* CRISPR/Cas9. N/TERTs were grown in KC-SFM medium (ThermoFisher #17005-042) supplemented with 30 μg/ml bovine pituitary extract, 0.2 ng/ml epidermal growth factor, and 0.3 mM calcium chloride.

### Generation of Myd88-KO KCs by CRISPR/Cas9

Single-guide RNA (sgRNA) target sequence was developed using a web interface for CRISPR design (http://crispr.mit.edu). The pSpCas9 (BB)-2A-GFP (PX458) was a gift from Feng Zhang (Addgene plasmid # 48138) and used as cloning backbone. We followed the CRISPR/Cas9 protocol from Ran et al. (54) to generate Myd88-KO KCs. In short, for the Myd88 knockout, the following oligonucleotides were used for annealing: MYD88sgRNA2F: 5′-CACCGCTGCTCTCAACATGCGAGTG-3′ and MYD88sg RNA2R: 5′-AAACCACTCGCATGTTGAGAGCAGC-3′. The annealed oligonucleotides were inserted into the cloning vector Px458 following the Ran et al. (54) protocol. Ligated plasmids were transformed into competent *E. coli* (ThermoFisher # C737303) and then plated on LB-agar plate overnight. Multiple colonies were selected for plasmid preparation (Qiagen # 27106), and Sanger sequencing validated a plasmid with proper insertion of Myd88 sgRNA target sequence. The plasmid with proper insertion was transfected into an immortalized KC line (N/TERTs) using the TransfeX transfection kit (ATCC # ACS4005). Single cells positive for GFP were sorted into 96-well plates using a MoFlo Astrios #1 cell sorter and grown up to ~50% confluency. Cells from 96-well plates were transferred into 12-well plates and grown to 50% confluency. DNA was extracted and PCR amplified using specific primers, MYD88SG1PCRF1: CTCCTCCACATCCTCC CTTC, MYD88SG1PCRR1: AGTTGCCGGATCTCCAAGTA. We selected KCs with homozygous mutation, which was validated by Sanger sequencing.

### Cytokine Treatment, RNA Extraction, and qRT-PCR

MYD88-KO KCs including WT KCs were grown in 12-well plates, and cells were treated with recombinant IL-1 beta (10 μg/ml; R&D Systems # 201-LB-025), IL-36 gamma (10 μg/ml; R&D Systems # 6835-IL-010), IFN-gamma (50 μg/ml; R&D Systems # 285-IF-100), IL-17A (20 μg/ml; R&D Systems # 317-ILB-050), and/or TNF-alpha (10 μg/ml; R&D Systems # 210-TA-005) for 8 or 24 h. RNAs were isolated from cell cultures using Qiagen RNeasy plus kit (Cat # 74136). Reversed transcription was performed using High Capacity cDNA Transcription kit (ThermoFisher # 4368813). qPCR was performed on a 7900HT Fast Real-time PCR System (ThermoFisher) with TaqMan Universal PCR Master Mix (ThermoFisher # 4304437) using TaqMan primers (ThermoFisher Scientific; *IL1B*: Hs01555410_m1, *IL36G*: Hs00219742_m1, *BIRC3*: Hs00985031_g1, *NFKB2*: Hs01028890_g1, *NFKBIA*: Hs00355671_g1, *CXCL8*: Hs00174103_m1, *S100A7*: Hs01923188_u1, and *TNFAIP3*: Hs00234713_m1). *RPLP0* (ThermoFisher # Hs99999902_m1) was used as a loading CTL.

### Small Interfering RNA (siRNA) Protocol for *NFKB1* and *ETS1* Knockdowns

Small interfering RNAs were purchased from ThermoFisher Scientific (*NFKB1* siRNA: Ambion Silencer Select siRNA cat no. 4392420, ID no. 9506, 2 μl of 2 μM stock per transfection; *ETS1* siRNA: Ambion Silencer siRNA cat no. AM16708, ID no. 115623, 2 μl of 2 μM stock per transfection; and SCR siRNA: Ambion Silencer Negative Control #1 siRNA cat no. AM4611, 0.8 μl of 5 μM stock per transfection). siRNA was combined with the P3 solution and layered over cells following the Lonza protocol. Cells were electroporated *via* Amaxa 4D-Nucleofector using Lonza Amaxa P3 Primary Cell 4D-Nucleofector X Kit S 32 reactions (cat no. V4XP-3032), which includes 2 × 16-well strips and P3 solution. The nucleofection program was optimized with guidance from Lonza technical support. Cells and siRNA were loaded into 16-well strips and pulsed as appropriate following the cell type electroporation program. Cells were then plated into 12-well plates (Corning Costar cat no. 3513) for 48 h to reach confluency, and cytokine treatment was administered for 8 h past nucleofection, after which total RNA was extracted using the Qiagen RNeasy Plus Mini kit (cat no. 74136). qPCR was performed as described earlier with TaqMan primers (see list of primers above; *NFKB1*: Hs00765730_m1; *ETS1*: Hs00428293_m1; *CXCL1*: Hs00236937_m1; *CXCL2*: Hs00601975_m1; *RELA*: Hs01042014_m1; *RELB*: Hs00232399_m1; and *DEFB4*: Hs00175474_m1).

## Results

### Whole Transcriptome Sequencing Analysis of Epidermal KCs Treated with IL-1 Family Cytokines (IL-1B, IL-36A, IL-36B, and IL-36G)

Whole transcriptome sequencing (RNA-seq) was used to profile expression of KCs treated with IL-1B, IL-36A, IL-36B, or IL-36G (8 or 24 h treatment; *n* = 2–3 samples per treatment and time point; *n* = 34 samples total). We obtained an average of 28.1 million reads per sample (26.0 million after QC filtering; Figures S1A,B in Supplementary Material). An average of 91.6% of reads mapped to the human genome, with 97.3% of mapped reads assigned to intragenic regions (Figures S1C,D in Supplementary Material). Expression profiling efficiency (% exonic reads) was 95% (Figure S1E in Supplementary Material), and expression of 12,917 protein-coding genes was detected on average among samples (Figure S1F in Supplementary Material).

Cluster analysis showed that KCs treated with cytokines for 8 h grouped with CTL samples (Figure S2A in Supplementary Material). However, KCs treated with cytokines for 24 h clustered apart from CTL samples, indicating stronger responses with extended cytokine treatment (Figure S2A in Supplementary Material). Consistent with this, when samples were plotted with respect to PC axes, 8 h cytokine and CTL samples overlapped, whereas 24 h cytokine and CTL samples did not (Figures S2C,D in Supplementary Material). Among protein-coding genes, treatment group (CTL, IL-1B, IL-36A, IL-36B, or IL-36G) and treatment time (8 or 24 h) explained a similar percentage of gene expression variation (Figure S2E in Supplementary Material). The cell line evaluated (lines A, B, or C derived from different donors) was the strongest factor explaining gene expression variation (Figure S2E in Supplementary Material).

### IL-1B and IL-36 Expression Responses Are More Disparate at 8 h of Treatment but Harmonize after 24 h

**Figure 1:**
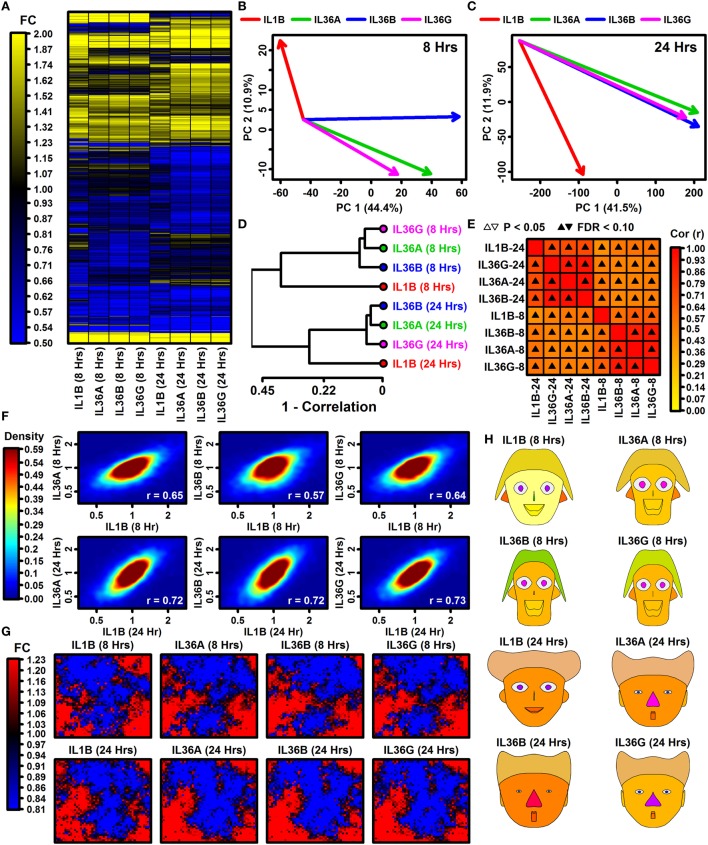
Global transcription responses to IL-1B and IL-36 (RNA-seq). **(A)** FC heatmap. The heatmap shows FC estimates [cytokine/control (CTL)] from 7,059 keratinocyte (KC)-expressed genes altered with respect to at least 1 cytokine (*P* < 0.05; FC > 1.50 or FC < 0.67). Hierarchical gene clustering was performed using average linkage and Euclidean distance. RNA-seq experiments were replicated with *n* = 2 or 3 samples per treatment. **(B,C)** Principal component (PC) response vectors. Arrows begin at the CTL sample bivariate PC mean and end at the bivariate PC mean for the indicated cytokine. **(D)** Cluster analysis. Cytokine responses were clustered based upon the expression changes for 13,752 KC-expressed genes (distance = 1 − Spearman rank correlation). **(E)** Correlations among cytokine responses (13,752 KC-expression genes; Spearman rank correlation). **(F)** Comparison of IL-36 cytokine responses to IL-1B. Colors reflect gene density within the scatterplot (see scale; bottom right: Spearman rank correlation). **(G)** Self-organizing maps (SOMs). The SOM layout was determined based upon expression patterns of all KC-expressed genes. Colors reflect the average FC estimate (cytokine/CTL) for genes assigned to a given SOM region. **(H)** Chernoff faces. Face features are scaled or color-coded to reflect cytokine responses for 15 selected genes (see Materials and Methods). The genes were chosen by algorithm as representatives of clusters with different IL-1B and IL-36 response profiles.

Global analyses suggested broadly similar expression responses to IL-1B and IL-36, although hierarchical clustering showed that a minority of genes exhibit cytokine- or time-specific responses (Figure 1A). Responses to IL-36A, IL-36B, and IL-36G varied more following 8 h of treatment but were more consistent after 24 h (Figures 1B,C). Correspondingly, IL-36 expression responses were more similar to those of IL-1B following 24 h of treatment (0.72 ≤ *r*_s_ ≤ 0.73) compared with 8 h of treatment (0.57 ≤ *r*_s_ ≤ 0.65) (Figure 1F). Consistent with these trends, SOMs and Chernoff faces reflective of genome-wide response patterns were more similar among cytokines at 24 h of treatment compared with 8 h (Figures 1G,H). These trends demonstrate that expression responses to IL-1B, IL-36A, IL-36B, and IL-36G vary more with early treatment (8 h) but partially harmonize with increased treatment time (24 h).

### Type I and II Interferon Genes Have Time-Dependent IL-1B and IL-36 Responses

We identified between 788 and 1,747 DEGs significantly altered by IL-1B, IL-36A, IL-36B, or IL-36G following 8 or 24 h of treatment (FDR < 0.10 with FC > 2.00 or FC < 0.50; Figure S3 in Supplementary Material). For each cytokine, a similar number of DEGs was identified following 8 or 24 h of treatment (Figures S3A–P in Supplementary Material), and overall expression responses at 8 h were modestly correlated with those at 24 h (0.44 ≤ *r*_s_ ≤ 0.52; Figure S3Q in Supplementary Material). However, it was possible to identify genes with time-dependent cytokine responses (FDR < 0.10; Figure S4A in Supplementary Material), including genes decreased at 8 h but increased at 24 h (*VIM, CHRNB1*, and *RPTN*), and genes increased at 8 h but decreased at 24 h (*IFIT1B, IFIT1*, and *IFIT2*) (Figures S4B,C in Supplementary Material). The largest number of time-dependent responses was observed in IL-36G-treated cells (Figures S4A,D,E in Supplementary Material). Genes decreased by IL-36G at 8 h but then increased at 24 h were associated with nucleic acid metabolism and transcription (Figure S4F in Supplementary Material). Conversely, genes increased by IL-36G at 8 h but decreased at 24 h were associated with interferon signaling and response to virus (Figure S4G in Supplementary Material).

**Figure 2:**
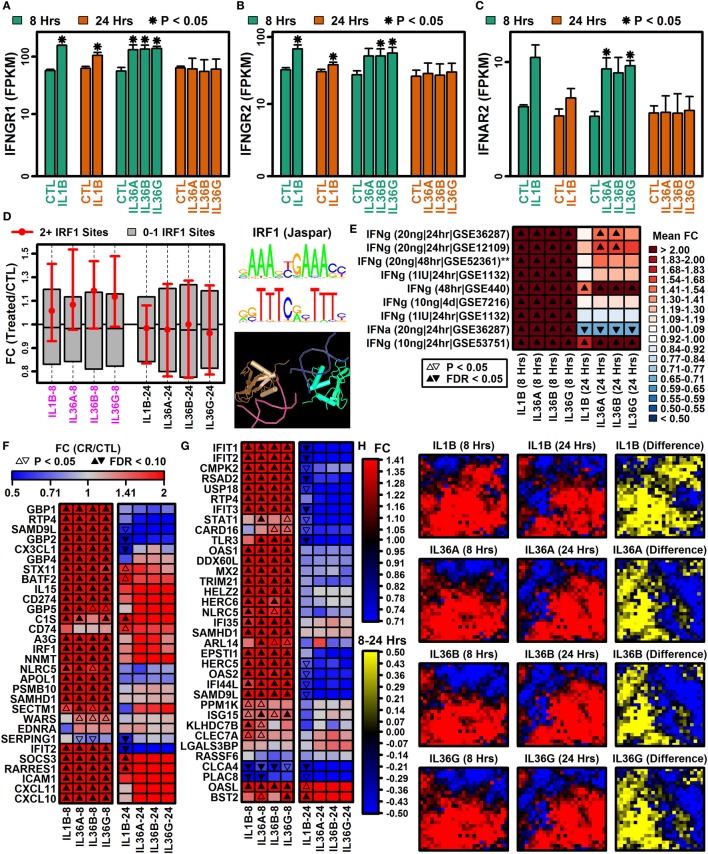
Type I and II interferon genes have time-dependent IL-1B and IL-36 responses. **(A)** Interferon gamma receptor 1 (*IFNGR1*). **(B)** Interferon gamma receptor 2 (*IFNGR2*). **(C)** Interferon alpha and beta receptor subunit 2 (*IFNAR2*). In panels **(A–C)**, average FPKM (±1 SE) is shown for each group, and asterisks denote significant differences relative to the control (CTL) treatment at the corresponding time point (paired two-sample *t*-test; *n* = 2 or 3 per treatment). **(D)** Genes with interferon response factor 1 (IRF1) binding sites (5 kb upstream region). The middle 50% of FC estimates is shown for genes with 2+ IRF1 binding sites compared with genes with fewer IRF1 sites (magenta font, horizontal axis: *P* < 0.05, Wilcoxon rank sum test). The IRF1 position weight matrix is shown (right) along with the IRF1 tetrameric structure (bottom right; NCBI structure database). **(E)** IFN-induced gene signature scores. IFN-induced genes were identified from microarray studies of IFN-treated keratinocytes (left margin), and the average FC for these genes was calculated in IL-1B/IL-36 experiments (bottom margin). Left margin labels indicate the cytokine concentration (in ml), treatment duration, and GEO series accession number. All cytokine experiments were replicated with at least two samples per treatment. **(F)** Top 30 IFN-g-induced genes (identified from GSE36287). **(G)** Top 35 INFa-induced genes (identified from GSE36287). **(H)** Self-organizing maps (SOMs). The SOM layout was determined only from IFN-g-induced genes (i.e., 2,500 genes most strongly induced by IFN-g, GSE36287). Colors reflect average FC estimates for IFN-g-induced genes assigned to each SOM region (columns 1 and 2 on left). The final column (yellow–blue) displays the mean FC difference for each cytokine with respect to each SOM region (8 h mean FC–24 h mean FC; log_2_ scale).

Genes encoding interferon receptor components were upregulated more strongly by IL-1B and IL-36 at 8 h compared with 24 h, e.g., interferon gamma receptor 1 (*IFNGR1*), interferon gamma receptor 2 (*IFNGR2*), and interferon alpha and beta receptor subunit 2 (*IFNAR2*) (Figures 2A–C). Consistent with this, genes with two or more interferon response factor 1 (*IRF1*) binding sites were more likely to be increased by IL-1B and IL-36 at 8 h of stimulation but not 24 h (Figure 2D). In addition, type I and II interferon-responsive genes identified by microarray in human KCs were more strongly elevated by IL-1B/IL-36 following 8 h of treatment compared with 24 h (Figures 2E–G). Of 2,500 genes most strongly induced by IFN-g in human KCs, most were more strongly elevated by IL-1B at 8 h compared with 24 h (Figure 2H). These results demonstrate time-dependent IFN responses to IL-1B and IL-36 in KCs, with early IFN gene induction (8 h) that fades or reverses to repression with increased treatment time (24 h).

### Early IL-1B-Specific Responses Are Associated with Epithelium Development and Mitosis

Responses to IL-1B and IL-36 cytokines differed more at 8 h compared with 24 h (Figure 1). At the 8 h time point, it was thus possible to identify some genes specifically induced by IL-1B but not IL-36 cytokines (e.g., *GRHL3, IL22RA1*, and *FOSL2*), and some genes repressed by IL-1B but not IL-36 cytokines (e.g., *CRIP1, TMEM200B*, and *CDK1*) (Figure S5 in Supplementary Material). Genes within the former group were significantly associated with epithelium development, morphogenesis, and regulation of gene expression (Figure S5E in Supplementary Material), while genes from this latter group were significantly associated with mitosis, cell division, and chromosome segregation (Figure S5F in Supplementary Material). At the 24 h time point, we identified fewer IL-1B-specific responses (Figure S5 in Supplementary Material). Likewise, most genes responding to the IL-36 cytokines showed similar trends with IL-1B treatment (Figure S6 in Supplementary Material). The small number of genes showing opposing responses included *VSIG8, GIMAP2, STC2*, and *PER1* (increased by IL-36 cytokines but not IL-1B), as well as *C22orf23, SLC9A3, FAM124B*, and *SLC46A2* (decreased by IL-36 cytokines but not IL-1B) (Figure S6 in Supplementary Material).

### Robust Expression Responses Consistent across Time and Shared by All Four Cytokine Treatments (IL-1B, IL-36A, IL-36B, and IL-36G)

**Figure 3:**
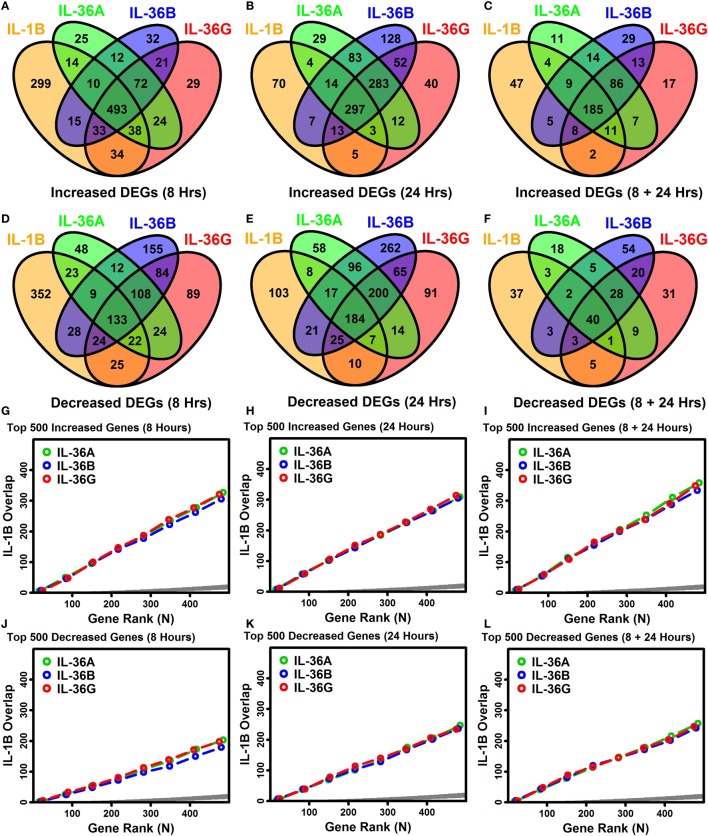
Overlap among IL-1B, IL-36A, IL-36B, and IL-36G transcriptional responses in keratinocytes. **(A–F)** Venn diagrams showing the number of differentially expressed genes (DEGs) altered by multiple cytokines as identified by RNA-seq **(A–C)** cytokine-increased DEGs; **(D–F)** cytokine-decreased DEGs. Venn diagrams in panels **(C,F)** show the number of DEGs similarly altered by cytokines at both time points. **(G–L)** Overlap of ranked gene lists (IL-1B vs. IL-36 cytokines). Genes most strongly increased **(G–I)** or decreased **(J–L)** by each cytokine treatment were ranked (based upon *P*-values), and the overlap is shown (vertical axis) with respect to varying gene ranks (top 1–500 genes, horizontal axis). An overlap above the dark gray region is greater than expected by chance (Fisher’s Exact Test, *P* < 0.05).

**Figure 4:**
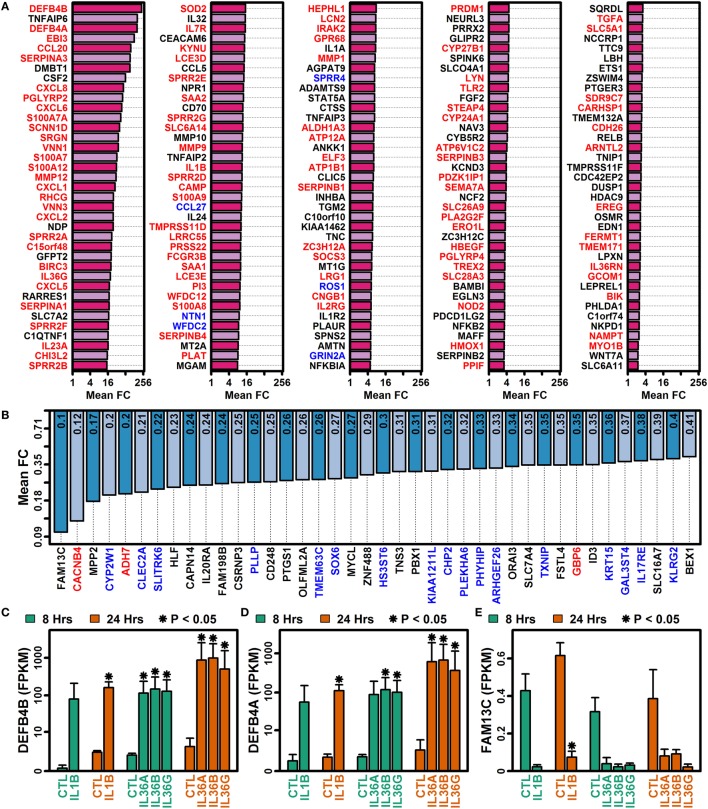
Genes altered by all four cytokines (IL-1B, IL-36A, IL-36B, and IL-36G) at both time points (8 and 24 h). **(A)** 185 IL-1B/IL-36-increased DEGs. **(B)** 40 IL-1B/IL-36-decreased DEGs. All genes listed in panels **(A,B)** were identified by RNA-seq analysis of cultured keratinocytes with *n* = 2 or 3 samples per treatment. Gene symbols with red labels (left margin) are significantly elevated in psoriasis lesions (PP/PN FC > 2.0, false discovery rate (FDR) < 0.10; RNA-seq meta-analysis), and genes with blue labels are significantly decreased in psoriasis lesions (PP/PN FC < 0.50, FDR < 0.10; RNA-seq meta-analysis). **(C)** Defensin beta 4B (*DEFB4B*). **(D)** Defensin beta 4A (*DEFB4A*). **(E)** Family with sequence similarity 13 member C (*FAM13C*). In panels **(C–E)**, average FPKM (±1 SE) is shown for each group, and asterisks denote significant differences relative to the control treatment at the corresponding time point (paired two-sample *t*-test; *n* = 2 or 3 per treatment).

IL-1B and IL-36 cytokines interact with different receptors although these share a common IL-1RAcP subunit that may permit cross-stimulation (1, 10). Among IL-36 cytokines, for example, 70–90% of genes regulated by one IL-36 cytokine were correspondingly altered by another at the same time point (Figures 3A–F). Many of these were similarly altered by IL-1B, and it was possible to identify genes with consistent responses to all four cytokines. Following 8 h of treatment, 493 DEGs were elevated, and 133 DEGs were repressed by all 4 cytokines (Figures 3A,D). Similarly, following 24 h of treatment, 297 DEGs were elevated, and 184 DEGs were repressed by all 4 cytokines, respectively (Figures 3A,D). Intersecting these results, we identified 185 DEGs elevated by all 4 cytokines at both time points, along with 40 DEGs repressed by all 4 cytokines at both time points (Figures 3C,F). These 225 DEGs (185 increased + 40 decreased) define a shared response of epidermal KCs to IL-1B, IL-36A, IL-36B, and IL-36G that is consistent at both early and late time points (Figure 4).

### Most IL-1B and IL-36-Responsive DEGs Identified by RNA-Seq Are Not Identified As Differentially Expressed in Previous Microarray Analyses

The gene expression response to IL-1B was previously evaluated in monolayer KC cultures (55), and the response to IL-36 has been evaluated in reconstituted human epidermis (56). Both studies used microarrays to quantify gene expression (55, 56). In the prior IL-36 study (56), the IL-36 concentration applied to KCs was 5 μg/ml, whereas in this study the concentration was more similar to that observed in human serum (10 ng/ml). Fold changes estimated by RNA-seq in this study showed positive but limited genome-wide correlation to FCs from these prior analyses (*r*_s_ ≤ 0.26), usually with increased correlations among genes with high expression compared with those with low expression (Figure S7A in Supplementary Material). Of the 225 DEGs altered by all 4 cytokines in our study (8 + 24 h), 180 had not been correspondingly altered in the prior IL-1B microarray analysis (Figures S7B,C in Supplementary Material). Our re-analysis of the prior IL-36 microarray study did not identify any DEGs at an FDR threshold of 0.10. Some genes had been altered twofold in the microarray study, but at least 140 of the 185 IL-1B/IL-36-increased DEGs had not been increased twofold, whereas at least 37 of the 40 IL-1B/IL-36-decreased DEGs had not been decreased twofold (Figures S7D–I in Supplementary Material). RNA-seq-increased DEGs with similar trends in the array analyses included *CCL20, S100A7A*, and *S100A7* (Figure S7J in Supplementary Material), while increased DEGs not altered or decreased in prior array datasets included *SERPINA3, DMBT1*, and *PGLYRP2* (Figure S7K in Supplementary Material). Likewise, RNA-seq-decreased DEGs trending toward decreased expression in array datasets included *PLLP, TMEM63C*, and *CHP2*, while RNA-seq-decreased DEGs not altered or increased in the array datasets included *SLC7A4, OLFML2A*, and *GBP6* (data not shown).

### IL-1B and IL-36 Amplify Cytokine/Chemokine Production and Genes Associated with Leukocyte Chemotaxis, Neutrophil Activation, and Mucosal Immunity

**Figure 5:**
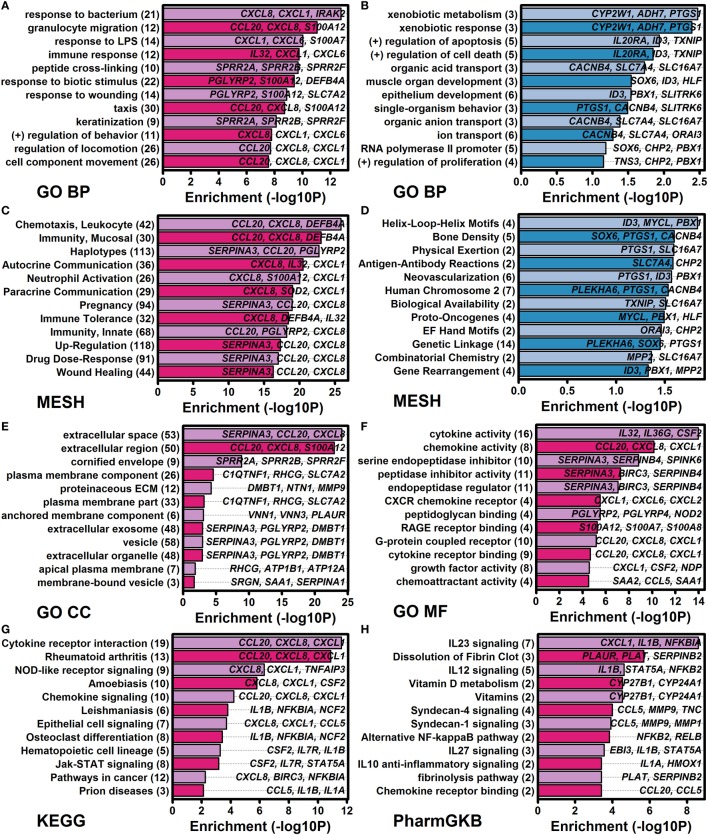
Functional analysis of genes altered by all four cytokines (IL-1B, IL-36A, IL-36B, and IL-36G) at both time points (8 and 24 h). Cytokine-regulated genes identified by RNA-seq were evaluated for functional enrichment using multiple gene annotation databases [GO, medical subject heading (MeSH), KEGG, and PharmGKB]. **(A)** GO BP terms enriched among 185 IL-1B/IL-36-increased differentially expressed genes (DEGs). **(B)** GO BP terms enriched among 40 IL-1B/IL-36-decreased DEGs. **(C)** MeSH terms enriched among increased DEGs. **(D)** MeSH terms enriched among decreased DEGs. **(E)** GO CC terms enriched among increased DEGs. **(F)** GO MF terms enriched among increased DEGs. **(G)** KEGG terms enriched among increased DEGs. **(H)** PharmGKB pathways enriched among increased DEGs. In panels **(A–H)**, the number of genes associated with each term is indicated in parentheses (left margin), and associated example genes are listed within each figure.

The 185 elevated DEGs included genes encoding cytokines (*IL1A, IL1B, IL23A, IL32*, and *IL36G*) and chemokines (*CCL20, CXCL1, CXCL2, CXCL5, CXCL6*, and *CXCL8*) (Figure 4A). Consistent with this, increased DEGs collectively showed significant associations with cytokine activity, chemokine activity, and cytokine receptor interaction (Figure 5); furthermore, a significant proportion of increased DEGs were localized to the extracellular space suggesting roles as secreted factors (Figure 5E). The 185 DEGs were also associated with gene categories related to leukocyte recruitment (e.g., granulocyte migration, taxis, leukocyte chemotaxis, neutrophil activation, and chemoattractant activity), mucosal immunity, and epithelial cell signaling (Figure 5). Compared with these findings, the 40 decreased DEGs exhibited weaker and less significant functional associations but were significantly associated with xenobiotic metabolism, positive regulation of apoptosis, development, and transport (Figures 5B,D). Increased and decreased DEGs were both associated with different aspects of bone or joint physiology (increased DEGs: rheumatoid arthritis, osteoclast differentiation, and vitamin D metabolism; decreased DEGs: bone density; Figure 5).

### Genes Induced by IL-1B and IL-36 Are Similarly Elevated in Skin Lesions from PsV, GPP, and Other Inflammatory Skin Diseases

**Figure 6:**
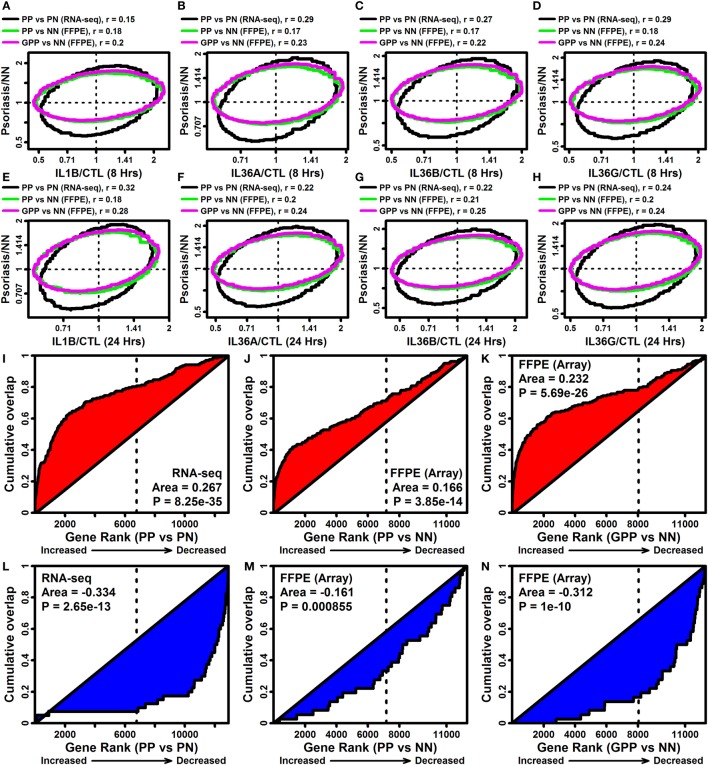
IL-1B/IL-36-regulated genes overlap significantly with genes altered in psoriasis lesions [PV and generalized pustular psoriasis (GPP)]. **(A–H)** FC comparison [cytokine/control (CTL) vs. lesional/NN]. FC estimates from IL-1B/IL-36-treated keratinocytes (RNA-seq) were compared with those obtained from the comparison between PP/GPP lesions and PN/NN skin. Comparisons were made to PP vs. PN FC estimates from an RNA-seq meta-analysis (*n* = 44 patients), and GPP/PN vs. NN FC estimates from a prior microarray study [GSE79704; formalin-fixed paraffin-embedded (FFPE) tissues; *n* = 12–20 per group]. Each ellipse outlines the 75% of genes closest to the bivariate mean based upon the Mahalanobis distance (top margin: spearman correlation coefficients). **(I–K)** Gene set enrichment analysis [185 IL-1B/IL-36-increased differentially expressed genes (DEGs)]. Genes were ranked according their expression change in PP or GPP lesions (horizontal axis), and the cumulative overlap of the 185 IL-1B/IL-36-increased DEGs is shown (vertical axis). **(L–N)** Gene set enrichment analysis (40 IL-1B/IL-36-decreased DEGs). Genes were ranked according their expression change in PP or GPP lesions (horizontal axis), and the cumulative overlap of the 40 IL-1B/IL-36-increased DEGs is shown (vertical axis). *P*-values in panels **(I–N)** are calculated using the Wilcoxon rank sum test.

Pro-inflammatory effects of IL-1B and IL-36 may contribute to cutaneous inflammation in psoriatic disease (18). Consistent with this possibility, expression responses to IL-1B and IL-36 in epidermal KCs were correlated with those in lesional psoriasis skin (Figures 6A–H). This analysis was performed with comparisons to expression responses in chronic plaque psoriasis (PsV) estimated from a recent RNA-seq meta-analysis (fresh frozen tissues), and with comparison to responses in PsV and GPP from microarray analysis of FFPE tissues (Figures 6A–H) (10). Each lesional skin signature was positively correlated with IL-1B and IL-36 responses in epidermal KCs, but in all cases the correlation was slightly stronger for GPP compared with PsV lesions (FFPE samples) (Figures 6A–H). In agreement with this trend, the 185 DEGs elevated by all 4 cytokines were more strongly enriched among GPP-increased genes compared with PsV-increased genes (FFPE samples; Figures 6I,J,K). Likewise, the 40 DEGs repressed by all 4 cytokines were more strongly enriched among GPP-decreased genes compared with PV-decreased genes (FFPE samples; Figures 6L,M,N).

Expression responses to IL-1B and IL-36 were additionally correlated with transcriptional alterations observed in other skin diseases, such as atopic dermatitis (*r*_s_ = 0.291), eczema (*r*_s_ = 0.283), and allergic dermatitis (*r*_s_ = 0.269) (Figure S8 in Supplementary Material). Lesions from these and other skin diseases tended to show elevated expression of IL-1B/IL-36-increased DEGs and decreased expression of IL-1B/IL-36-decreased DEGs (e.g., acne, eschars, and *H. ducreyi* infection; Figure S9A in Supplementary Material). Approximately 90% of the 185 IL-1B/IL-36-increased DEGs, for example, were expressed at higher levels in acne lesions compared with normal skin (Figure S9C in Supplementary Material), while 70% of the 40 IL-1B/IL-36-decreased genes were expressed at lower levels in acne lesions (Figure S9F in Supplementary Material).

### Genes Induced by IL-1B and IL-36 Overlap Significantly with Genes Near GWAS Loci Linked to Autoimmune and Autoinflammatory Conditions (IBD, Psoriasis, and PsA)

**Figure 7:**
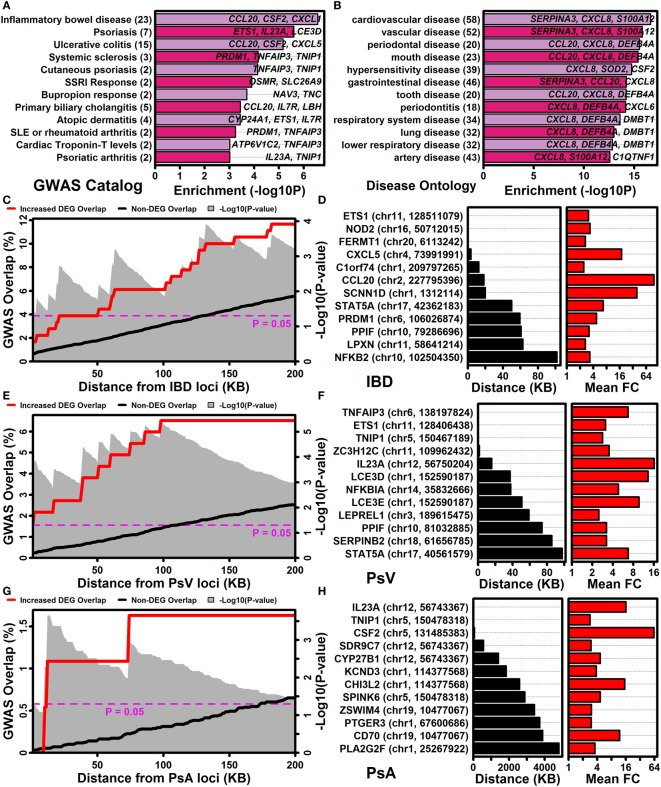
IL-1B/IL-36-regulated genes are located near GWAS loci associated with inflammatory bowel disease, psoriasis vulgaris, and psoriatic arthritis. **(A)** NHGRI-EBI GWAS catalog. Diseases or traits linked to genes by the GWAS catalog were evaluated for overlap with IL-1B/IL-36-increased differentially expressed genes (DEGs) (Fisher’s Exact Test). **(B)** Disease ontology database. Diseases or traits linked to genes by the disease ontology database were evaluated for overlap with IL-1B/IL-36-increased DEGs (Fisher’s Exact Test). In panels **(A,B)**, the number of disease/trait-associated DEGs is listed in parentheses (left margin). **(C)** Percent overlap (left axis) between the 185 IL-1B/IL-36-increased DEGs and genes at varying distances (bottom axis) from inflammatory bowel disease (IBD)-associated SNPs. The magnitude of overlap was compared between increased DEGs and non-DEGs (gray background, Fisher’s Exact Test). **(D)** Increased DEGs closest to IBD-associated SNPs. The SNP location is listed for each DEG (left margin). **(E–H)** The analyses shown in panels **(C,D)** were repeated with respect to GWAS loci associated with psoriasis vulgaris (PsV) and psoriatic arthritis (PsA).

The NHGRI-EBI GWAS catalog (47) was used to determine if IL-1B/IL-36-increased DEGs overlapped significantly with genes linked to various human diseases or traits (Figure 7A). This identified multiple autoimmune and/or autoinflammatory conditions for which associated genes overlapped significantly with IL-1B/IL-36-increased DEGs, including IBD/ulcerative colitis, psoriasis/PsA, systemic sclerosis, primary biliary cholangitis, atopic dermatitis, lupus, and rheumatoid arthritis (Figure 7A). These trends were reinforced by analyses of genes linked to diseases based upon the Disease Ontology database (57), which revealed that increased DEGs were associated with gastrointestinal disease, diseases involving hypersensitivity reactions, and respiratory disease (Figure 7B).

The association between increased DEGs and IBD was stronger than any other observed with respect to the NHGRI-EBI GWAS catalog (Figure 7A), with approximately 12% of IL-1B/IL-36-increased DEGs located within 200 kb of an IBD-associated locus (Figure 7C). Increased DEGs closest to an IBD-associated locus included *ETS1, NOD2*, and *FERMT1* (Figure 7D). Genes near loci linked to PsV and PsA by GWA studies also overlapped significantly with increased DEGs (Figures 7A,E,G). Increased DEGs closest to a PsV locus included *TNFAIP3, ETS1, TNIP*, and *ZC3H12C* (Figure 7F), while increased DEGs closest to a PsA locus included *IL23A, TNIP1*, and *CSF2* (Figure 7H).

### The IL-1B and IL-36 Transcriptional Response Rebalances Pathway Activation through Positive and Negative Feedback

IL-1B and IL-36 treatment of epidermal KCs induced expression of mRNAs encoding the ligands *IL1A, IL1B, IL36A, IL36B*, and *IL36G* (Figure S10A in Supplementary Material), demonstrating that these cytokines have the ability to positively regulate their own expression. We also noted increased expression of cathepsin S (*CTSS*), which cleaves and activates IL-36 cytokines (Figure S10A in Supplementary Material), along with elevated expression of proteins encoding receptor subunits (*IL1RAP*) or downstream signaling elements (*IRAK2, MYD88*, and *TAB2*) (Figure S10A in Supplementary Material). Some transcription responses were associated with inhibitory negative feedback, however, with increased expression of genes encoding receptor antagonists (*IL1RN* and *IL36RN*), anti-inflammatory ligands (*IL1F10* and *IL37*), and a decoy receptor (*IL1R2*); furthermore, we observed decreased expression of some signaling proteins (*TAB1* and *IRAK1BP1*) (Figure S10A in Supplementary Material). These expression shifts of IL-1 family-associated genes could in some cases be replicated by other cytokine treatments applied to epidermal KCs (e.g., TNF, IFN-g, and IL-17A; Figures S10B–D in Supplementary Material). Potentially, this was due to induction of *IL1B* and *IL36G* mRNAs by these other cytokines, yielding an expression response closely matching that of IL-1B or IL-36 (Figure S10A in Supplementary Material). The 40 IL-1B/IL-36-decreased DEGs, for example, were almost uniformly repressed by IL-17A treatment of KCs in a prior microarray study (Figure S10E in Supplementary Material).

### NF-KappaB and ETS1 Transcription Factor Binding Sites Are Enriched in Regions Upstream of Genes Induced by IL-1B and IL-36

A dictionary of 2,934 motifs known to interact with vertebrate transcription factors and uDBPs was screened to identify motifs enriched in 5,000 bp regions upstream of IL-1B/IL-36-responsive genes (16). We identified 185 motifs significantly enriched in regions 5,000 bp upstream of the 185 IL-1B/IL-36-increased DEGs (FDR < 0.10), along with 5 motifs enriched in regions upstream of the 40 IL-1B/IL-36-decreased DEGs (FDR < 0.10).

The 185 motifs identified with respect to IL-1B/IL-36-increased DEGs were frequently associated with 5-GGAA/TTCC-3 and 5-AGTC/GACT-3 elements recognized by TFs from the immunoglobulin fold or basic domain superfamilies (Figure S11A in Supplementary Material). There was additionally strong overrepresentation of motifs associated with TFs from the ETS, SOX, GATA, JUN, FOS, and NF-kappaB families (Figure S11B in Supplementary Material). Of the 3 most significantly enriched motifs, 2 interacted with NF-kappaB (Figures S11C,D in Supplementary Material) and consistent with this the 185 IL-1B/IL-36-increased DEGs included 2 genes encoding NF-kappaB subunit proteins (i.e., *NFKB2* and *RELB*; Figure 4A; Figure S11F in Supplementary Material). Several increased DEGs belonged to the NF-kappaB signaling pathway (KEGG pathway hsa04064), including upstream regulators (*IL1B, LYN*, and *BIR3*) and downstream targets regulated transcriptionally by pathway activation (*TNFAIP3, NFKBIA, CXCL2*, and *CXCL8*) (Figure S12 in Supplementary Material). Motifs known to interact with AP-1 were also significantly enriched in regions upstream of increased DEGs (FDR = 1.45e−4) although were not included among the top-ranked motifs (Figure S11C in Supplementary Material).

The NF-kappaB motif most strongly enriched among increased DEGs featured a 5-GGAA/TTCC-3 half site (Figure S11D in Supplementary Material) that was prominent among the complete set of 185 motifs enriched among increased DEG upstream regions (Figure S11A in Supplementary Material). Notably, this half site resembled an ETS1 binding site included in our dictionary (Figure S11E in Supplementary Material), which was also significantly enriched among increased DEG upstream regions (Figure S11C in Supplementary Material). Consistent with this result, *ETS1* was among the 185 IL-1B/IL-36-increased DEGs (Figure 3A; Figure S11G in Supplementary Material).

### IL-1B and IL-36G Expression Responses Are MyD88 Dependent

**Figure 8:**
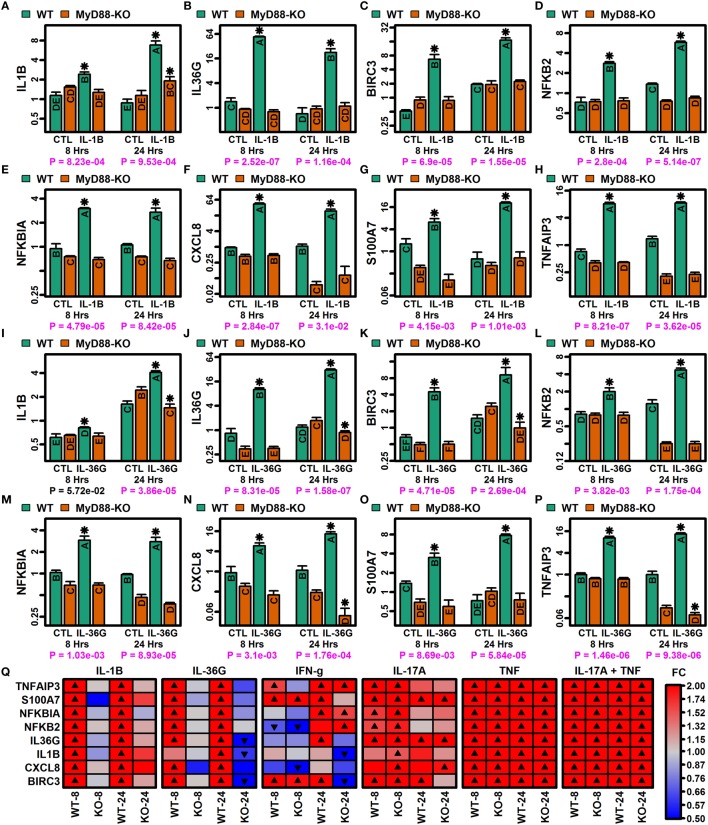
MyD88-dependent expression responses to IL-1B and IL-36G (RT-PCR). CRISPR/Cas9 was used to generate MyD88-knockout (KO) N/TERT immortalized keratinocytes (KCs) (*n* = 3 per treatment). **(A–H)** IL-1B expression responses in WT and MyD88-KO KCs. (**I–P)** IL-36G expression responses in WT and MyD88-KO KCs. In panels **(A–P)**, groups without the same letter are significantly different (*P* < 0.05, Fisher’s least significant difference). An asterisk is used to denote cytokine treatments that differ significantly from their corresponding control (*P* < 0.05, Fisher’s least significant difference). *P*-values in the bottom margin are derived from linear models testing for a treatment-by-genotype interaction effect. **(Q)** Heatmaps showing expression responses to IL-1B, IL-36G, IFN-g, IL-17A, TNF, and IL-17A + TNF in WT and MyD88-KO KCs. Expression of ribosomal protein lateral stalk subunit P0 (*RPLP0*) was used as an internal reference in all analyses **(A–Q)**.

Myeloid differentiation primary response gene 88 (*MYD88*) encodes an adaptor protein mediating NF-kappaB activation downstream of IL-1B receptor stimulation (Figure S12 in Supplementary Material). Since NF-kappaB binding sites were enriched in regions upstream of genes induced by IL-1B and IL-36, we used CRISPR/Cas9 to generate MyD88-KO KCs and investigated whether MyD88-KO and WT KCs exhibited differential responses to IL-1B and IL-36G stimulation (Figure 8). We evaluated expression responses of eight IL-1B/IL-36-increased DEGs, including mRNAs encoding IL-1B and IL-36G (*IL1B* and *IL36G*), predicted NF-kappaB target genes (*TNFAIP3, NFKBIA, NFKB2, CXCL8*, and *BIRC3*) (Figure S12B in Supplementary Material), and psoriasin (*S100A7*). All eight genes showed differential responses in MyD88-KO and WT KCs, with upregulation of mRNA expression following stimulation with IL-1B or IL-36G in WT KCs, but no induction in MyD88-KO KCs (Figures 8A–P). These response patterns were observed only with IL-1B and IL-36G stimulation of WT and KO KCs, with both genotypes showing similar expression responses following stimulation by IFN-g, IL-17A, TNF, and IL-17A + TNF (Figure 8Q).

### Loss of NF-KappaB and ETS1 Function Modulates IL-36 Target Gene Expression but Does Not Abrogate Expression Responses to IL-36G Stimulation

The 185 IL-1B/IL-36-increased DEGs had a significantly increased frequency of NF-kappaB and ETS1 motifs in their upstream regions (Figure S11 in Supplementary Material). We therefore used siRNA to knockdown expression of *NFKB1* (p50) and *ETS1* by approximately 75% or greater in non-stimulated and IL-36-treated KCs (Figure S13 in Supplementary Material). To some degree, *NFKB1* knockdown modified expression responses to IL-36G stimulation for some IL-36 target genes, including *TNFAIP3, CXCL2*, and *RELB* (*P* < 0.05, treatment-by-siRNA interaction effect; Figure S13 in Supplementary Material). However, for most IL-36 targets, neither *NFKB1* nor *ETS1* knockdown abrogated the expression response to IL-36G stimulation (Figure S14 in Supplementary Material). While not altering the expression response, IL-36G target genes were nonetheless sensitive to the siRNA treatments in CTL or IL-36G-treated cells, respectively. For instance, *NFKB1* knockdown decreased expression of *BIRC3, NFKB2*, and *RELB* in CTL or IL-36G-treated cells (*P* < 0.05; Figure S14 in Supplementary Material). However, in most cases, *NFKB1* and *ETS1* knockdown increased expression of IL-36 target genes (*NFKB1* siRNA: *IL1B, CXCL8, TNFAIP3, CXCL1, CXCL2*, and *RELA*; *ETS1* siRNA: *IL1B, IL36G, BIRC3, S100A7, CXCL1, CXCL2*; *P* < 0.05; Figure S14 in Supplementary Material).

## Discussion

The cytokines IL-36A, IL-36B, and IL-36G are members of the IL-1 family that have increasingly been linked to autoimmune and autoinflammatory diseases affecting epithelial tissues (2). These diseases include psoriasis (PsV and GPP) but have recently expanded to include acute generalized exanthematous pustulosis and autoimmune blistering diseases (e.g., dermatitis herpetiformis, bullous pemphigoid, and pemphigus vulgaris) (58, 59). To better understand the role of IL-36 in these conditions, we used RNA-seq to evaluate effects of IL-1B and IL-36 in primary epidermal KCs. We identified early IL-1B-specific responses involving genes contributing to epidermal development and mitosis, along with time-dependent IL-1B and IL-36 responses of type I and II interferon genes. There was strong overlap among responses to all four cytokines with shared responses of genes near GWAS loci linked to autoimmune/autoinflammatory conditions, including PsV, PsA, IBD, primary biliary cholangitis, and systemic sclerosis. CRISPR/Cas9 mutagenesis demonstrated that shared IL-1B/IL-36 responses depend completely upon MyD88 adaptor protein. Using the broad lens of RNA-seq, these results provide a global and comprehensive view of IL-1B and IL-36 responses in a disease-relevant epithelial cell type. The robust gene sets defined by our study provide points of reference for future analyses, and our findings highlight gene-level connections between autoimmune/autoinflammatory diseases and an MyD88-dependent pathway activated downstream of IL-1B and IL-36.

IL-36 receptor includes an IL-1RAcP subunit that also binds IL-1B and may contribute to overlapping IL-36 and IL-1B responses in epidermal KCs (1). Indeed, most genes regulated by IL-36 cytokines were correspondingly regulated by IL-1B (Figure 3). Following 8 h of cytokine stimulation, it was possible to identify some DEGs regulated only by IL-1B and not IL-36 cytokines (Figures S5A,B in Supplementary Material), but after 24 h of stimulation almost all IL-1B regulated genes were correspondingly altered by IL-36 cytokines (Figures S5C,D in Supplementary Material). The highly similar transcriptional responses of the IL-36 cytokines we examined, IL-36A, IL-36B, and IL-36G, are even more remarkable (Figure 3). These findings prompt the question of why IL-1B and three distinct IL-36 cytokines are necessary when their transcriptional fingerprints are so redundant. Although potentially deleterious in the settings of autoimmune and autoinflammatory diseases, IL-1B and IL-36 have critical roles in immune response and pathogen defense (60, 61). This was apparent from our data since genes induced by IL-1B and IL-36 were above all else associated with response to bacterium/LPS and granulocyte migration (Figure 5A). From this perspective, functional redundancy may be advantageous, particularly considering that IL-1B and IL-36 cytokines may not all be expressed within the same tissue. Moreover, whereas IL-1B may be active when secreted, IL-36 cytokines require extracellular processing to gain full activation, which may influence the rate at which downstream responses can develop and contribute to pathogen defense (1). Functional redundancies suggested by our analyses may thus represent an important feature enabling more rapid defense responses, which would be especially critical for epithelial tissues in which IL-36 cytokines are most highly expressed (60, 61).

IL-36 cytokines are generally regarded as pro-inflammatory and may act to promote KC and immune cell activation during acute stages of psoriatic lesion development (18). In agreement with this, our findings reveal several ways in which IL-36 amplifies the cytokine network in epidermal KCs, such as upregulation of stimulatory ligands (*IL1A, IL1B, IL36A, IL36B*, and *IL36G*), activating proteases (*CTSS*), and key signaling components (*IL1RAP, IRAK2, MYD88*, and *TAB2*). In these respects, the IL-36 signaling pathway exhibits feed-forward dynamics with the capacity to self-amplify following initial stimulation. Upregulation of cathepsin S (*CTSS*) by IL-1B and all IL-36 cytokines is particularly significant, since cathepsin S was recently identified as an activating protease for IL-36 (62) and it had been observed that cathepsin S is selectively expressed by psoriatic KCs (63). Since other proteases capable of activating IL-36 cytokines are neutrophil-derived (20), *CTSS* upregulation would provide a mechanism for self-amplification of the IL-36 cascade independent of inflammatory cell infiltration into psoriatic lesions. Despite these effects, IL-1B and IL-36 cannot be regarded as strictly pro-inflammatory, since both cytokines also upregulated anti-inflammatory ligands (*IL36RN, IL1RN, IL37*, and *IL1F10*) and downregulated some signaling components (*IRAK1BP1* and *TAB1*). These changes in gene expression may represent compensatory responses that rebalance the IL-36 signaling system to limit excessive or prolonged activation, and their role in psoriatic or other autoimmune diseases may be equally significant, as suggested by the association between GPP and IL-36RN polymorphisms (64). Our findings thus reinforce the idea that IL-36 signaling depends on the balance of several components within an integrated system, with net activation dependent upon the cumulative actions of all components, including extracellular proteases, cytokine ligands, receptor subunits, and downstream signaling elements (24).

The cytokines most convincingly demonstrated to mediate psoriasis plaque formation are those for which inhibition has been effective in patients, such as IL-23, TNF, and IL-17A (9). Although these cytokines lie outside of the IL-1 family, our findings reveal connections to IL-1B and IL-36 signaling in each case, with the IL-1B/IL-36 cascade functioning as either an upstream regulator or downstream target. In the case of IL-23, our results showed that both IL-1B and IL-36 increased expression of *IL23A* mRNA, demonstrating that anti-IL-23 therapies target a protein positively regulated by the IL-1B/IL-36 signaling cascade. This effect on *IL23A* expression was additionally notable as a connection between the IL-1B/IL-36 target gene set and those genes linked by GWA studies to PsV and PsA (Figures 7F,H). On the other hand, we identified several cytokine treatments in epidermal KCs leading to a transcriptional response similar to that observed for IL-1B and IL-36 (e.g., IFN-g, TNF, and IL-17A; Figure S10 in Supplementary Material), and further inspection revealed that these treatments upregulate expression of *IL1A, IL1B*, and *IL36G* mRNAs (Figure S10A in Supplementary Material). IL-1B and IL-36 thus function not only as an important regulator of disease-mediating cytokines (i.e., *IL23A*) but also as a downstream target of such cytokines (i.e., *TNF* and *IL17A*). These results are further supported by studies demonstrating co-localization of IL-36A staining with IL-17 and IL-23 in epidermal sections of GPP and acute generalized exanthematous pustulosis (58), correlated expression of IL-36 cytokines, IL-17A, and TNF in psoriasis lesions (65), and an association between IL-36A and IL-17 levels in serum of patients with autoimmune blistering diseases (59).

The shared genetic basis of autoimmune diseases has long been supported by clustering of diverse autoimmune diseases within certain families (i.e., familial autoimmunity) (66). The role of IL-36 in multiple types of autoimmune and autoinflammatory disease is not completely understood, but so far evidence has linked IL-36 to psoriasis (PsV, PsA, and GPP), rheumatoid arthritis, IBD, systemic lupus erythematosus, and Sjögren’s syndrome (2, 7). In our study, a significant proportion of genes elevated by IL-1B and IL-36 belonged to the KEGG rheumatoid arthritis pathway (e.g., *CCL20, CXCL7*, and *CXCL1*; Figure 5). In agreement with this, IL-36A and IL-36B abundance were significantly elevated in serum of rheumatoid arthritis patients compared with healthy CTLs (8), and IL-36A was also elevated in synovial tissues from rheumatoid arthritis patients as compared with osteoarthritis patients (17). When considering genes linked to diseases based only upon GWAS findings, we observed significant overlap between IL-1B/IL-36-increased genes and genes linked to PsV (e.g., *TNFAIP3, ETS1, TNIP1*, and *ZC3H12C*) and PsA (e.g., *IL23A, TNIP1*, and *CSF2*) (Figure 7). Surprisingly, however, the strongest overall association we observed based upon GWAS findings was between IL-1B/IL-36-increased genes and IBD (Figure 7A), with a total of 23 increased genes linked to IBD based upon GWAS findings, including *ETS1, NOD2, FERMT1*, and *CXCL5* (Figure 7D). The two main manifestations of IBD include Th1-mediated Crohn’s disease and Th2-mediated ulcerative colitis, and of these we observed a much stronger association to GWAS findings for ulcerative colitis (*P* = 6.7 × 10^−6^) compared with Crohn’s disease (*P* = 0.16). Consistent with this, recent studies have demonstrated stronger elevation of mRNAs encoding IL-36 cytokines in the inflamed mucosa of ulcerative colitis patients compared with Crohn’s disease (6). In addition to these findings, our results demonstrate significant overlap between IL-1B/IL-36 increased DEGs and genes linked by GWA studies to systemic sclerosis and primary biliary cholangitis (Figure 7A). The role of IL-36 has not been as well studied in these autoimmune conditions, but our findings justify investigations to better understand the significance of IL-36 signaling in these contexts.

IL-36 receptor stimulation is expected to recruit MyD88 adaptor protein and subsequently activate JNK/ERK pathways, with downstream transcriptional responses coordinated by NF-kappaB and AP-1 (6, 19, 21, 22). This model of IL-36 action has largely been established from studies of transformed cell lines rather than primary cells from disease-relevant tissues (19). In primary epidermal KCs, our findings support dependence of IL-36 responses on MyD88 activity, with abrogation of IL-36 responses in the absence of functional MyD88 (Figure 8). Downstream of MyD88, IL-1B, and IL-36 stimulation increased expression of genes encoding NF-kappaB subunits (*NFKB2* and *RELB*), and we observed enrichment of NF-kappaB binding sites upstream of IL-36-increased DEGs. We identified similar enrichment of AP-1 binding sites with respect to IL-36-increased DEGs (data not shown), although genes encoding AP-1 subunits were not included among the 185 genes most robustly upregulated by IL-1B and IL-36 treatment (Figure 4A). Activation of NF-kappaB has been observed in PsV skin lesions and is positively correlated with abundance of each IL-36 cytokine (23). The role of NF-kappaB in psoriatic disease has been further demonstrated by associations with *CARD14* gain-of-function mutations, which lead to NF-kappaB activation and have been linked to PsV, PsA, and GPP (67). It is therefore surprising that siRNA knockdown of *NFKB1* (p50) expression did not inhibit IL-36 responses in epidermal KCs, even though expression of IL-1B/IL-36-sensitive genes was altered by *NFKB1* inhibition (Figure S14 in Supplementary Material). It is possible that NF-kappaB-dependent responses to IL-36 can proceed even in the setting of decreased *NFKB1* expression, in part due to compensatory collateral responses to *NFKB1* knockdown (68), such as increased *RELA* and *NFKB2* expression (Figures S14D,K in Supplementary Material) or increased endogenous production of IL-1B and IL-36G to yield stronger pathway stimulation (Figures S14A,B in Supplementary Material). Alternatively, although we demonstrated significant siRNA knockdown of *NFKB1* expression (Figure S13 in Supplementary Material), it is possible that residual protein abundance remained and was sufficient to permit IL-1B/IL-36 responses. Future studies using CRISPR/Cas9 mutagenesis of NFKB1 or other NF-kappaB subunits will therefore be valuable to further dissect out the role of this transcription factor in the IL-1B/IL-36-MyD88 signaling cascade.

The success of anti-cytokine therapies for chronic plaque psoriasis has raised hope that this approach can be extended to other cytokine targets for new drug development (9). Existing biologics have been remarkably effective but still do not resolve symptoms for all patients and for some patients drug effectiveness can decline over time (9). There thus remains a need for continuing research toward new treatments to incorporate into psoriasis patient care. We here demonstrate unique effects of IL-36 in epidermal KCs and functional associations between IL-36 and other cytokines validated as psoriasis drug targets (i.e., TNF, IL-17A, and IL-23). Our findings thus help to support a rationale for further studies to evaluate the translational potential of IL-36 for drug development in the setting of psoriatic disease and with respect to a broad range of autoimmune and/or autoinflammatory conditions (69).

## Ethics Statement

This study was carried out in accordance with the recommendations of the United States National Institutes of Health with written informed consent from all subjects. All subjects gave written informed consent in accordance with the Declaration of Helsinki. The protocol was approved by the University of Michigan institutional review board (Ann Arbor, MI, IRB No. HUM00037994).

## Author Contributions

WS analyzed the data. WS, MB, MS, and JG drafted the manuscript. MB, MS, SL, JF, and XX performed wet lab experiments; NW, LT, MK, and YL assisted in drafting the manuscript and revising it critically. All the authors have read and approved of the final manuscript.

## Conflict of Interest Statement

The authors declare that the research was conducted in the absence of any commercial or financial relationships that could be construed as a potential conflict of interest. The reviewer JA and handling editor declared their shared affiliation.

## References

[1] Garlanda C Dinarello CA Mantovani A. The interleukin-1 family: back to the future. Immunity (2013) 39(6):1003–18. 10.1016/j.immuni.2013.11.010 24332029 PMC3933951

[2] Hahn M Frey S Hueber AJ. The novel interleukin-1 cytokine family members in inflammatory diseases. Curr Opin Rheumatol (2017) 29(2):208–13. 10.1097/bor.0000000000000361 27926540

[3] Blumberg H Dinh H Trueblood ES Pretorius J Kugler D Weng N Opposing activities of two novel members of the IL-1 ligand family regulate skin inflammation. J Exp Med (2007) 204(11):2603–14. 10.1084/jem.20070157 17908936 PMC2118475

[4] Tortola L Rosenwald E Abel B Blumberg H Schafer M Coyle AJ Psoriasiform dermatitis is driven by IL-36-mediated DC-keratinocyte crosstalk. J Clin Invest (2012) 122(11):3965–76. 10.1172/jci63451 23064362 PMC3484446

[5] Scheibe K Backert I Wirtz S Hueber A Schett G Vieth M IL-36R signalling activates intestinal epithelial cells and fibroblasts and promotes mucosal healing in vivo. Gut (2017) 66(5):823–38. 10.1136/gutjnl-2015-310374 26783184

[6] Nishida A Hidaka K Kanda T Imaeda H Shioya M Inatomi O Increased expression of interleukin-36, a member of the interleukin-1 cytokine family, in inflammatory bowel disease. Inflamm Bowel Dis (2016) 22(2):303–14. 10.1097/mib.0000000000000654 26752465

[7] Ciccia F Accardo-Palumbo A Alessandro R Alessandri C Priori R Guggino G Interleukin-36alpha axis is modulated in patients with primary Sjogren’s syndrome. Clin Exp Immunol (2015) 181(2):230–8. 10.1111/cei.12644 25902739 PMC4516438

[8] Wang M Wang B Ma Z Sun X Tang Y Li X Detection of the novel IL-1 family cytokines by QAH-IL1F-1 assay in rheumatoid arthritis. Cell Mol Biol (Noisy-le-grand) (2016) 62(4):31–4. 10.14715/cmb/2016.62.4.6 27188731

[9] Veilleux MS Shear NH. Biologics in patients with skin diseases. J Allergy Clin Immunol (2017) 139(5):1423–30. 10.1016/j.jaci.2017.03.012 28477721

[10] Johnston A Xing X Wolterink L Barnes DH Yin Z Reingold L IL-1 and IL-36 are dominant cytokines in generalized pustular psoriasis. J Allergy Clin Immunol (2017) 140(1):109–20. 10.1016/j.jaci.2016.08.056 28043870 PMC5494022

[11] Onoufriadis A Simpson MA Pink AE Di Meglio P Smith CH Pullabhatla V Mutations in IL36RN/IL1F5 are associated with the severe episodic inflammatory skin disease known as generalized pustular psoriasis. Am J Hum Genet (2011) 89(3):432–7. 10.1016/j.ajhg.2011.07.022 21839423 PMC3169817

[12] Marrakchi S Guigue P Renshaw BR Puel A Pei XY Fraitag S Interleukin-36-receptor antagonist deficiency and generalized pustular psoriasis. N Engl J Med (2011) 365(7):620–8. 10.1056/NEJMoa1013068 21848462

[13] Mahil SK Twelves S Farkas K Setta-Kaffetzi N Burden AD Gach JE AP1S3 mutations cause skin autoinflammation by disrupting keratinocyte autophagy and up-regulating IL-36 production. J Invest Dermatol (2016) 136(11):2251–9. 10.1016/j.jid.2016.06.618 27388993 PMC5070969

[14] Liang Y Sarkar MK Tsoi LC Gudjonsson JE. Psoriasis: a mixed autoimmune and autoinflammatory disease. Curr Opin Immunol (2017) 49:1–8. 10.1016/j.coi.2017.07.007 28738209 PMC5705427

[15] D’Erme AM Wilsmann-Theis D Wagenpfeil J Holzel M Ferring-Schmitt S Sternberg S IL-36gamma (IL-1F9) is a biomarker for psoriasis skin lesions. J Invest Dermatol (2015) 135(4):1025–32. 10.1038/jid.2014.532 25525775

[16] Swindell WR Sarkar MK Stuart PE Voorhees JJ Elder JT Johnston A Psoriasis drug development and GWAS interpretation through in silico analysis of transcription factor binding sites. Clin Transl Med (2015) 4:13. 10.1186/s40169-015-0054-5 25883770 PMC4392043

[17] Frey S Derer A Messbacher ME Baeten DL Bugatti S Montecucco C The novel cytokine interleukin-36alpha is expressed in psoriatic and rheumatoid arthritis synovium. Ann Rheum Dis (2013) 72(9):1569–74. 10.1136/annrheumdis-2012-202264 23268368

[18] Towne JE Sims JE. IL-36 in psoriasis. Curr Opin Pharmacol (2012) 12(4):486–90. 10.1016/j.coph.2012.02.009 22398321

[19] Towne JE Garka KE Renshaw BR Virca GD Sims JE. Interleukin (IL)-1F6, IL-1F8, and IL-1F9 signal through IL-1Rrp2 and IL-1RAcP to activate the pathway leading to NF-kappaB and MAPKs. J Biol Chem (2004) 279(14):13677–88. 10.1074/jbc.M400117200 14734551

[20] Henry CM Sullivan GP Clancy DM Afonina IS Kulms D Martin SJ. Neutrophil-derived proteases escalate inflammation through activation of IL-36 family cytokines. Cell Rep (2016) 14(4):708–22. 10.1016/j.celrep.2015.12.072 26776523

[21] Takahashi K Nishida A Shioya M Imaeda H Bamba S Inatomi O Interleukin (IL)-1beta is a strong inducer of IL-36gamma expression in human colonic myofibroblasts. PLoS One (2015) 10(11):e0138423 10.1371/journal.pone.0138423 26562662 PMC4643060

[22] Zhu L Wu Y Wei H Yang S Zhan N Xing X Up-regulation of IL-23 p19 expression in human periodontal ligament fibroblasts by IL-1beta via concurrent activation of the NF-kappaB and MAPKs/AP-1 pathways. Cytokine (2012) 60(1):171–8. 10.1016/j.cyto.2012.05.016 22688014

[23] He Q Chen HX Li W Wu Y Chen SJ Yue Q IL-36 cytokine expression and its relationship with p38 MAPK and NF-kappaB pathways in psoriasis vulgaris skin lesions. J Huazhong Univ Sci Technolog Med Sci (2013) 33(4):594–9. 10.1007/s11596-013-1164-1 23904383

[24] Walsh PT Fallon PG. The emergence of the IL-36 cytokine family as novel targets for inflammatory diseases. Ann N Y Acad Sci (2016). 10.1111/nyas.13280 27783881

[25] Elder JT Fisher GJ Zhang QY Eisen D Krust A Kastner P Retinoic acid receptor gene expression in human skin. J Invest Dermatol (1991) 96(4):425–33. 10.1111/1523-1747.ep12469889 1848877

[26] Martin M Cutadapt removes adapter sequences from high-throughput sequencing reads. EMBnetjournal (2011) 17:10–2. 10.14806/ej.17.1.200

[27] Hannon GJ FASTX-Toolkit Cold Spring Harbor Laboratory. (2009). Available from: http://hannonlab.cshl.edu/fastx_toolkit/

[28] Andrews S FastQC: A Quality Control Tool for High Throughput Sequence Data Babraham Bioinformatics. Babraham Bioinformatics (2015). Available from: http://www.bioinformatics.babraham.ac.uk/projects/fastqc/

[29] Kim D Pertea G Trapnell C Pimentel H Kelley R Salzberg SL. TopHat2: accurate alignment of transcriptomes in the presence of insertions, deletions and gene fusions. Genome Biol (2013) 14(4):R36. 10.1186/gb-2013-14-4-r36 23618408 PMC4053844

[30] Li H Handsaker B Wysoker A Fennell T Ruan J Homer N The sequence alignment/map format and SAMtools. Bioinformatics (2009) 25(16):2078–9. 10.1093/bioinformatics/btp352 19505943 PMC2723002

[31] Anders S Pyl PT Huber W HTSeq—a python framework to work with high-throughput sequencing data. Bioinformatics (2015) 31(2):166–9. 10.1093/bioinformatics/btu638 25260700 PMC4287950

[32] Trapnell C Roberts A Goff L Pertea G Kim D Kelley DR Differential gene and transcript expression analysis of RNA-seq experiments with TopHat and Cufflinks. Nat Protoc (2012) 7(3):562–78. 10.1038/nprot.2012.016 22383036 PMC3334321

[33] Wang L Wang S Li W. RSeQC: quality control of RNA-seq experiments. Bioinformatics (2012) 28(16):2184–5. 10.1093/bioinformatics/bts356 22743226

[34] DeLuca DS Levin JZ Sivachenko A Fennell T Nazaire MD Williams C RNA-SeQC: RNA-seq metrics for quality control and process optimization. Bioinformatics (2012) 28(11):1530–2. 10.1093/bioinformatics/bts196 22539670 PMC3356847

[35] Swindell WR Michaels KA Sutter AJ Diaconu D Fritz Y Xing X Imiquimod has strain-dependent effects in mice and does not uniquely model human psoriasis. Genome Med (2017) 9(1):24. 10.1186/s13073-017-0415-3 28279190 PMC5345243

[36] Toronen P Kolehmainen M Wong G Castren E. Analysis of gene expression data using self-organizing maps. FEBS Lett (1999) 451(2):142–6. 10.1016/S0014-5793(99)00524-4 10371154

[37] Chernoff H The use of faces to represent points in K-dimensional space graphically. J Am Stat Assoc (1973) 68(342):361–8. 10.1080/01621459.1973.10482434

[38] Robinson MD Oshlack A. A scaling normalization method for differential expression analysis of RNA-seq data. Genome Biol (2010) 11(3):R25. 10.1186/gb-2010-11-3-r25 20196867 PMC2864565

[39] Robinson MD McCarthy DJ Smyth GK. edgeR: a bioconductor package for differential expression analysis of digital gene expression data. Bioinformatics (2010) 26(1):139–40. 10.1093/bioinformatics/btp616 19910308 PMC2796818

[40] Landau WM Liu P. Dispersion estimation and its effect on test performance in RNA-seq data analysis: a simulation-based comparison of methods. PLoS One (2013) 8(12):e81415. 10.1371/journal.pone.0081415 24349066 PMC3857202

[41] Benjamini Y Hochberg Y Controlling the false discovery rate: a powerful and practical approach to multiple testing. J R Stat Soc B (1995) 57:289–300.

[42] Falcon S Gentleman R. Using GOstats to test gene lists for GO term association. Bioinformatics (2007) 23(2):257–8. 10.1093/bioinformatics/btl567 17098774

[43] Luo W Brouwer C. Pathview: an R/bioconductor package for pathway-based data integration and visualization. Bioinformatics (2013) 29(14):1830–1. 10.1093/bioinformatics/btt285 23740750 PMC3702256

[44] Tsuyuzaki K Morota G Ishii M Nakazato T Miyazaki S Nikaido I. MeSH ORA framework: R/bioconductor packages to support MeSH over-representation analysis. BMC Bioinformatics (2015) 16:45. 10.1186/s12859-015-0453-z 25887539 PMC4343279

[45] Yu G Wang LG Yan GR He QY. DOSE: an R/bioconductor package for disease ontology semantic and enrichment analysis. Bioinformatics (2015) 31(4):608–9. 10.1093/bioinformatics/btu684 25677125

[46] Swindell WR Remmer HA Sarkar MK Xing X Barnes DH Wolterink L Proteogenomic analysis of psoriasis reveals discordant and concordant changes in mRNA and protein abundance. Genome Med (2015) 7(1):86. 10.1186/s13073-015-0208-5 26251673 PMC4527112

[47] Welter D MacArthur J Morales J Burdett T Hall P Junkins H The NHGRI GWAS catalog, a curated resource of SNP-trait associations. Nucleic Acids Res (2014) 42(Database issue):D1001–6. 10.1093/nar/gkt1229 24316577 PMC3965119

[48] Stuart PE Nair RP Tsoi LC Tejasvi T Das S Kang HM Genome-wide association analysis of psoriatic arthritis and cutaneous psoriasis reveals differences in their genetic architecture. Am J Hum Genet (2015) 97(6):816–36. 10.1016/j.ajhg.2015.10.019 26626624 PMC4678416

[49] Swindell WR Johnston A Xing X Little A Robichaud P Voorhees JJ Robust shifts in S100a9 expression with aging: a novel mechanism for chronic inflammation. Sci Rep (2013) 3:1215. 10.1038/srep01215 23386971 PMC3564041

[50] Wingender E Schoeps T Donitz J. TFClass: an expandable hierarchical classification of human transcription factors. Nucleic Acids Res (2013) 41(Database issue):D165–70. 10.1093/nar/gks1123 23180794 PMC3531165

[51] Irizarry RA Hobbs B Collin F Beazer-Barclay YD Antonellis KJ Scherf U Exploration, normalization, and summaries of high density oligonucleotide array probe level data. Biostatistics (2003) 4(2):249–64. 10.1093/biostatistics/4.2.249 12925520

[52] Smyth GK. Linear models and empirical bayes methods for assessing differential expression in microarray experiments. Stat Appl Genet Mol Biol (2004) 3:Article3. 10.2202/1544-6115.1027 16646809

[53] Dickson MA Hahn WC Ino Y Ronfard V Wu JY Weinberg RA Human keratinocytes that express hTERT and also bypass a p16(INK4a)-enforced mechanism that limits life span become immortal yet retain normal growth and differentiation characteristics. Mol Cell Biol (2000) 20(4):1436–47. 10.1128/MCB.20.4.1436-1447.2000 10648628 PMC85304

[54] Ran FA Hsu PD Wright J Agarwala V Scott DA Zhang F. Genome engineering using the CRISPR-Cas9 system. Nat Protoc (2013) 8(11):2281–308. 10.1038/nprot.2013.143 24157548 PMC3969860

[55] Sa SM Valdez PA Wu J Jung K Zhong F Hall L The effects of IL-20 subfamily cytokines on reconstituted human epidermis suggest potential roles in cutaneous innate defense and pathogenic adaptive immunity in psoriasis. J Immunol (2007) 178(4):2229–40. 10.4049/jimmunol.178.11.7487-a 17277128

[56] Johnston A Xing X Guzman AM Riblett M Loyd CM Ward NL IL-1F5, -F6, -F8, and -F9: a novel IL-1 family signaling system that is active in psoriasis and promotes keratinocyte antimicrobial peptide expression. J Immunol (2011) 186(4):2613–22. 10.4049/jimmunol.1003162 21242515 PMC3074475

[57] Kibbe WA Arze C Felix V Mitraka E Bolton E Fu G Disease ontology 2015 update: an expanded and updated database of human diseases for linking biomedical knowledge through disease data. Nucleic Acids Res (2015) 43(Database issue):D1071–8. 10.1093/nar/gku1011 25348409 PMC4383880

[58] Song HS Kim SJ Park TI Jang YH Lee ES. Immunohistochemical comparison of IL-36 and the IL-23/Th17 axis of generalized pustular psoriasis and acute generalized exanthematous pustulosis. Ann Dermatol (2016) 28(4):451–6. 10.5021/ad.2016.28.4.451 27489427 PMC4969474

[59] Zebrowska A Wozniacka A Juczynska K Ociepa K Waszczykowska E Szymczak I Correlation between IL36alpha and IL17 and activity of the disease in selected autoimmune blistering diseases. Mediators Inflamm (2017) 2017:8980534 10.1155/2017/8980534 28611508 PMC5458385

[60] Winkle SM Throop AL Herbst-Kralovetz MM IL-36gamma augments host defense and immune responses in human female reproductive tract epithelial cells. Front Microbiol (2016) 7:955 10.3389/fmicb.2016.00955 27379082 PMC4911402

[61] Ahsan F Moura-Alves P Guhlich-Bornhof U Klemm M Kaufmann SH Maertzdorf J Role of interleukin 36gamma in host defense against tuberculosis. J Infect Dis (2016) 214(3):464–74. 10.1093/infdis/jiw152 27389350

[62] Ainscough JS Macleod T McGonagle D Brakefield R Baron JM Alase A Cathepsin S is the major activator of the psoriasis-associated proinflammatory cytokine IL-36gamma. Proc Natl Acad Sci U S A (2017) 114(13):E2748–57. 10.1073/pnas.1620954114 28289191 PMC5380102

[63] Schonefuss A Wendt W Schattling B Schulten R Hoffmann K Stuecker M Upregulation of cathepsin S in psoriatic keratinocytes. Exp Dermatol (2010) 19(8):e80–8. 10.1111/j.1600-0625.2009.00990.x 19849712

[64] Sugiura K Takemoto A Yamaguchi M Takahashi H Shoda Y Mitsuma T The majority of generalized pustular psoriasis without psoriasis vulgaris is caused by deficiency of interleukin-36 receptor antagonist. J Invest Dermatol (2013) 133(11):2514–21. 10.1038/jid.2013.230 23698098

[65] Carrier Y Ma HL Ramon HE Napierata L Small C O’Toole M Inter-regulation of Th17 cytokines and the IL-36 cytokines in vitro and in vivo: implications in psoriasis pathogenesis. J Invest Dermatol (2011) 131(12):2428–37. 10.1038/jid.2011.234 21881584

[66] Cardenas-Roldan J Rojas-Villarraga A Anaya JM. How do autoimmune diseases cluster in families? A systematic review and meta-analysis. BMC Med (2013) 11:73. 10.1186/1741-7015-11-73 23497011 PMC3655934

[67] Akiyama M Takeichi T McGrath JA Sugiura K Autoinflammatory keratinization diseases. J Allergy Clin Immunol (2017) 140(6):1545–47. 10.1016/j.jaci.2017.05.019 28668225

[68] Oda K Kitano H. A comprehensive map of the toll-like receptor signaling network. Mol Syst Biol (2006) 2:2006.0015. 10.1038/msb4100057 16738560 PMC1681489

[69] Wolf J Ferris LK. Anti-IL-36R antibodies, potentially useful for the treatment of psoriasis: a patent evaluation of WO2013074569. Expert Opin Ther Pat (2014) 24(4):477–9. 10.1517/13543776.2014.881473 24456078

