# Supplementary material for: RNA-Seq Analysis of IL-1B and IL-36 Responses in Epidermal Keratinocytes Identifies a Shared MyD88-Dependent Gene Signature
Source: Front Immunol. 2018 Jan 29;9:80. doi: 10.3389/fimmu.2018.00080 (PMC5796909; doi:10.3389/fimmu.2018.00080)

<u>Supplemental Figures</u>

RNA-seq analysis of IL-1B and IL-36 responses in epidermal keratinocytes identifies a shared MyD88-dependent gene signature

William R. Swindell[1*], Maria A. Beamer[2], Mrinal K. Sarkar[2], Shannon Loftus[2], Joseph Fullmer[2], Xianying Xing[2], Nicole L. Ward[3], Lam C. Tsoi[2], Michelle J. Kahlenberg[4], Yun Liang[2], Johann E. Gudjonsson[2]

[1]Ohio University, Heritage College of Osteopathic Medicine, Athens, OH, 45701 USA.
[2]University of Michigan, Department of Dermatology, Ann Arbor, MI, 48109, USA.
[3]Case Western Reserve University, Department of Dermatology, Cleveland, OH, 44106, USA.
[4]University of Michigan, Department of Internal Medicine, Division of Rheumatology, Ann Arbor, MI, 48109, USA.

[*]Correspondence:
Corresponding Author
ws277814@ohio.edu

Supplemental Figure 1. RNA-seq mapping and quality CTL. (A) Number of reads per sample prior to quality CTL filtering. (B) Number of reads per sample after quality CTL filtering. (C) Percentage of reads mapping to the genome. (D) Percentage of mapped reads assigned to intragenic regions. (E) Expression profiling efficiency (percentage of mapped reads assigned to exons). (F) Number of genes with detectable expression.

Supplemental Figure 2. Cluster and principal component analyses. (A) Cluster analysis. Samples were clustered based upon expression of protein-coding genes (FPKM; average linkage clustering based upon Euclidean distance). (B) Principal component plot. Samples are plotted with respect to the first 2 principal component axes. (C) Principal component plot (8 hour samples). (D) Principal component plot (24 hour samples). (E) Variable importance. Linear models were generated for each gene with cell line (A, B or C), time (8 or 24 hours), and treatment (CTL, IL-1B, IL-36A, IL-36B or IL-36G) as explanatory factors. Importance of each factor was assessed using likelihood ratio tests (LRTs). Boxes span the middle 50% of –log10-transformed p-values from LRTs for each factor (whiskers: 10th and 90th percentiles).

Supplemental Figure 3. Differential expression analyses. (A – D) Volcano plots (8 hours). (E – H) Volcano plots (24 hours). (I – L) MA plots (8 hours). (M – P) MA plots (24 hours). (Q) Scatterplot comparison of 8 and 24 hour expression responses to each cytokine. In (A – H), log10-transformed p-values (vertical axis) are plotted relative to FC estimates (horizontal axis) (red: cytokine-increased DEGs; blue: cytokine-decreased DEGs). In (I – P), FC estimates (vertical axis) are plotted relative to average expression for each gene (i.e., CPM or count per million mapped reads; red: cytokine-increased DEGs; blue: cytokine-decreased DEGs). In (Q), FC estimates are compared for all protein-coding genes with detectable expression and colors represent gene density. The estimated Spearman rank correlation estimate is shown (lower right).

Supplemental Figure 4. Time-dependent expression responses to IL-1B, IL-36A, IL-36B and/or IL-36G. (A) Number of genes exhibiting significant treatment-by-time interaction effects (FDR < 0.10; 8 hr. FC < 0.67 with 24 hr. FC > 1.50; 8 hr. FC > 1.50 with 24 hr. FC < 0.67). (B) Vimentin (*VIM*). (C) Interferon induced protein with tetratricopeptide repeats 2 (*IFIT2*). In (B) and (C), average FPKM (± 1 SE) is shown for each group and asterisks denote significant differences relative to the CTL treatment at the corresponding time point (paired two-sample t-test; n = 2 or 3 per group). (D) IL-36G time-dependent expression responses (▼ → ▲). (E) IL-36G time-dependent expression responses (▲ → ▼). (F) GO BP terms enriched among 30 genes with time-dependent responses to IL-36G (▼ → ▲). (G) GO BP terms enriched among 53 genes with time-dependent responses to IL-36G (▲ → ▼). In (F) and (G), the number of genes associated with each term is indicated in parentheses (left margin) and associated example genes with the strongest treatment-by-time interaction effects are listed (right margin).

Supplemental Figure 5. Genes with the most IL-1B-specific expression responses. (A) Top 25 genes most specifically up-regulated by 8 hours IL-1B treatment. (B) Top 25 genes most specifically down-regulated by 8 hours IL-1B treatment. (C) Top 25 genes most specifically up-regulated by 24 hours IL-1B treatment. (D) Top 25 genes most specifically down-regulated by 24 hours IL-1B treatment. (E – H) GO BP terms enriched among genes shown in (A) – (D), respectively. The number of genes associated with each term is indicated in parentheses (left margin) and associated example genes are listed within each figure.

Supplemental Figure 6. Genes with the most IL-36-specific expression responses. (A) Top 25 genes most specifically up-regulated by 8 hours IL-36 treatment. (B) Top 25 genes most specifically down-regulated by 8 hours IL-36 treatment. (C) Top 25 genes most specifically up-regulated by 24 hours IL-36 treatment. (D) Top 25 genes most specifically down-regulated by 24 hours IL-36 treatment. (E – H) GO BP terms enriched among genes shown in (A) – (D), respectively. The number of genes associated with each term is indicated in parentheses (left margin) and associated example genes are listed within each figure.

Supplemental Figure 7. RNA-seq identifies new IL-1B/IL-36-regulated genes not identified by previous microarray studies (GSE7216 and GSE25400). (A) RNA-seq versus microarray correlation of FC estimates (cytokine/CTL). Heatmap color densities correspond to gene density within each region (lower right: Spearman rank correlations based upon all genes; top margin: correlations based upon 50% of genes with lowest or highest FPKM). (B – I) Venn diagrams (RNA-seq versus microarray). RNA-seq DEGs include only those significantly altered by each cytokine at both time points (FDR < 0.10 with FC > 2.0 or FC < 0.50). Array DEGs include those altered significantly by IL-1B (FDR < 0.10 with FC > 2.0 or FC < 2.0) or genes altered 2-fold by IL-36A, IL-36B or IL-36G (*). Only genes with detectable expression in relevant samples from both platforms are included in each comparison. (J) IL-1B/IL-36-increased DEGs trending towards increased expression in previous microarray datasets. (K) IL-1B/IL-36-decreased DEGs unaltered or decreased in previous microarray datasets. In (J) and (K), black bars denote RNA-seq FC estimates while color-coded lines represent array-based estimates (see legend).

Supplemental Figure 8. KC expression responses to IL-1B/IL-36 are positively correlated with human skin disease signatures. (A) Cluster analysis. IL-1B/IL-36 responses (---) and human skin disease signatures were clustered (distance = 1 – *r*, *r* = Spearman correlation between signed log10-transformed p-values). Groups of correlated signatures are denoted by separate colors. (B) Spearman correlation matrix similarity matrix. The matrix shows correlation estimates among IL-1B/IL-36 expression responses and the 15 most strongly correlated disease signatures. (C – F) Highest Spearman correlation estimates. Expression responses are quantified based upon signed log10-transformed p-values (positive values: elevated expression in IL1B/IL36-treated KCs or human skin disease compared to normal skin; negative values: repressed expression in IL1B/IL36-treated KCs or human skin disease compared to normal skin). The robust regression estimate is shown (red line) with Spearman correlation estimate (lower right) (yellow ellipse: 90% of data points closest to bivariate mean based upon the Mahalanobis distance).

Supplemental Figure 9. Genes significantly altered by IL-1B/IL-36 are correspondingly altered in acne and other human skin diseases. (A) Skin diseases with elevated expression of the 185 IL-1B/IL-36-increased DEGs and decreased expression of the 40 IL-1B/IL-36-decreased DEGs. Expression in each skin disease was compared to uninvolved skin from patients (*) or normal skin from CTL subjects, with expression changes quantified using signed log10-transformed p-values (positive values: elevated expression in diseased skin; negative values: decreased expression in diseased skin). Symbols represent the median log10-p-value for IL-1B/IL-36-increased (red) and IL-1B/IL-36-decreased (blue) genes, respectively (error bars: 25th

to 75th percentile; grey boxes). Grey boxes represent the middle 50% of log10-p-values among all other genes. P-values (right margin) were generated by comparing log10-p-values between the 185 IL-1B/IL-36-increased DEGs and 40 IL-1B/IL-36-decreased DEGs (Wilcoxon rank sum test). (B) Gene set enrichment analyses (acne, GSE53795). Genes were ranked according their expression change in acne lesions (horizontal axis) and the cumulative overlap of the 185 IL-1B/IL-36-increased DEGs is shown (vertical axis). The p-value is calculated using the Wilcoxon rank sum test. (C) Acne FC estimates for the 185 IL-1B/IL-36-increased DEGs. (D) Overlap between acne-increased DEGs (FC > 2.00, FDR < 0.10) and the 185 IL-1B/IL-36-increased DEGs (excluding genes not measured/expressed in both experiments). (E, F, G) The 40 IL-1B/IL-36-decreased DEGs were analyzed in the same fashion as shown in (B) – (D). (H) IL-1B/IL-36-increased DEGs most strongly elevated in acne lesions (red font, bottom margin: FDR < 0.10, FC > 2.0). (I) IL-1B/IL-36-decreased DEGs most strongly decreased in acne lesions (blue font, bottom margin: FDR < 0.10, FC < 0.50).

Supplemental Figure 10. IL-1 family gene expression responses to cytokine treatment in epidermal KCs. (A) IL-1 family genes. Rows show gene expression changes in KCs treated with IL-1B/IL-36 (top rows; RNA-seq), psoriasis skin lesions (middle), or other cytokines (bottom; microarray results from previous experiments). (B) KC cytokine treatments leading to increased expression of the 185 IL-1B/IL-36-increased DEGs and decreased expression of the 40 IL-1B/IL-36-decreased DEGs. Expression changes are quantified using signed log10-transformed p-values (positive values: cytokine-increased expression; negative values: cytokine-decreased expression). In (A) and (B), labels from previous microarray studies (left margin) indicate the cytokine concentration (per mL), duration of cytokine treatment, and GEO series accession number (* = HaCaT; ** = reconstituted epidermis). All cytokine microarray experiments were performed with at least $n = 2$ replicates per treatment. (C, D) Gene set enrichment analyses (IL-17A response, GSE36287). Genes were ranked according to their expression change in IL-17A-treated KCs (horizontal axis) and the cumulative overlap of (C) increased DEGs or (D) decreased DEGs is shown (vertical axis) (p-value: Wilcoxon rank sum test). (E) IL-17A/CTL FC estimates for the 40 IL-1B/IL-36-decreased DEGs.

Supplemental Figure 11. NF-kappaB and ETS1 motifs are enriched in TSS-proximal regions upstream of IL-1B/IL-36-increased DEGs. (A) Cluster analysis of 185 motifs enriched in TSS-proximal regions of IL-1B/IL-36-increased DEGs (FDR < 0.10). Motifs were clustered using k-mer scores leading to the identification of motif groups with partial preference for 5-GAAA/TTTC-3 and 5-GTCA/TGAC-3 elements, respectively (yellow-black heatmap). Enrichment scores quantify the degree to which motifs resemble archetypes for different DNA-binding domain superfamily and class groups (red-black heatmaps). (B) TF families most frequently associated with the 185 motifs (right margin: Fisher's Exact Test p-value). (C) Motifs most strongly enriched among IL-1B/IL-36-increased DEGs. (D) NF-kappaB motif most strongly enriched among regions upstream of IL-1B/IL-36-increased DEGs. (E) ETS1 motif most strongly enriched among regions upstream of IL-1B/IL-36-increased DEGs. (F) Nuclear factor kappa B subunit 2 (*NFKB2*). (G) ETS proto-oncogene 1 (*ETS1*). In (F) and (G), average FPKM (± 1 SE) is shown for each group and asterisks denote significant differences relative to the CTL treatment at the corresponding time point (paired two-sample t-test).

Supplemental Figure 12. IL-1B/IL-36 induces expression of genes within the NF-kappaB signaling pathway (KEGG: hsa04064). (A) NF-kappaB pathway genes most strongly altered by IL-1B/IL-36. Genes shown in red font (top margin) were significantly increased by IL-1B/IL-36 in all treatments and both time points. (B) NF-kappaB signaling pathway. Pathway elements in red correspond to genes elevated by IL-1B/IL-36 in all treatments and both time points.

Supplemental Figure 13. Knockdown of *NFKB1* and *ETS1* expression using siRNA (RT-PCR). *NFKB1* or *ETS1* expression was knocked down using siRNA in CTL or IL-36-treated KCs (n = 3 per group; 8 hours cytokine treatment). Expression of ribosomal protein lateral stalk subunit P0 (*RPLP0*) was used as an internal reference in (A) and (B). Groups without the same letter are significantly different ($P < 0.05$, Fisher's least significant difference). Asterisks are used to denote cytokine treatments differing significantly from their corresponding control ($P < 0.05$, Fisher's least significant difference). P-values in the bottom margin address whether effects of cytokine treatment differed in CTL compared to siRNA cells (i.e., treatment-by-siRNA interaction effect; magenta font: $P < 0.05$).

Supplemental Figure 14. NFKB1- and ETS1-dependent expression in IL-36G-treated keratinocytes (RT-PCR). *NFKB1* or *ETS1* expression was knocked down using siRNA in CTL or IL-36-treated KCs (n = 3 per group; 8 hours cytokine treatment). Expression of ribosomal protein lateral stalk subunit P0 (*RPLP0*) was used as an internal reference in all analyses (A – L). Groups without the same letter are significantly different ($P < 0.05$, Fisher's least significant difference). P-values in the bottom margin address whether effects of cytokine treatment differed in CTL compared to siRNA cells (i.e., treatment-by-siRNA interaction effect; magenta font: $P < 0.05$).

Supplemental Figure 1

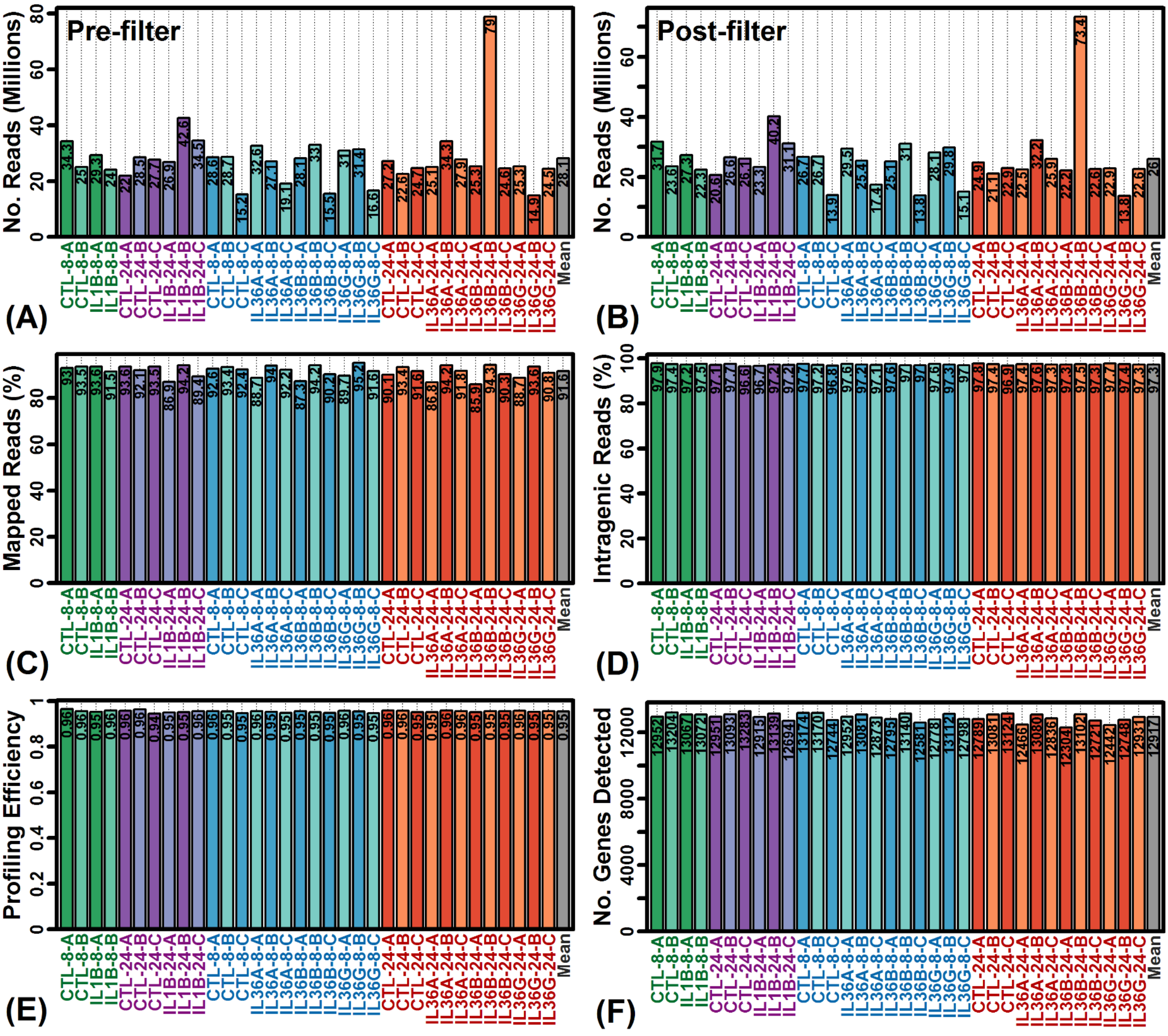

Supplemental Figure 2

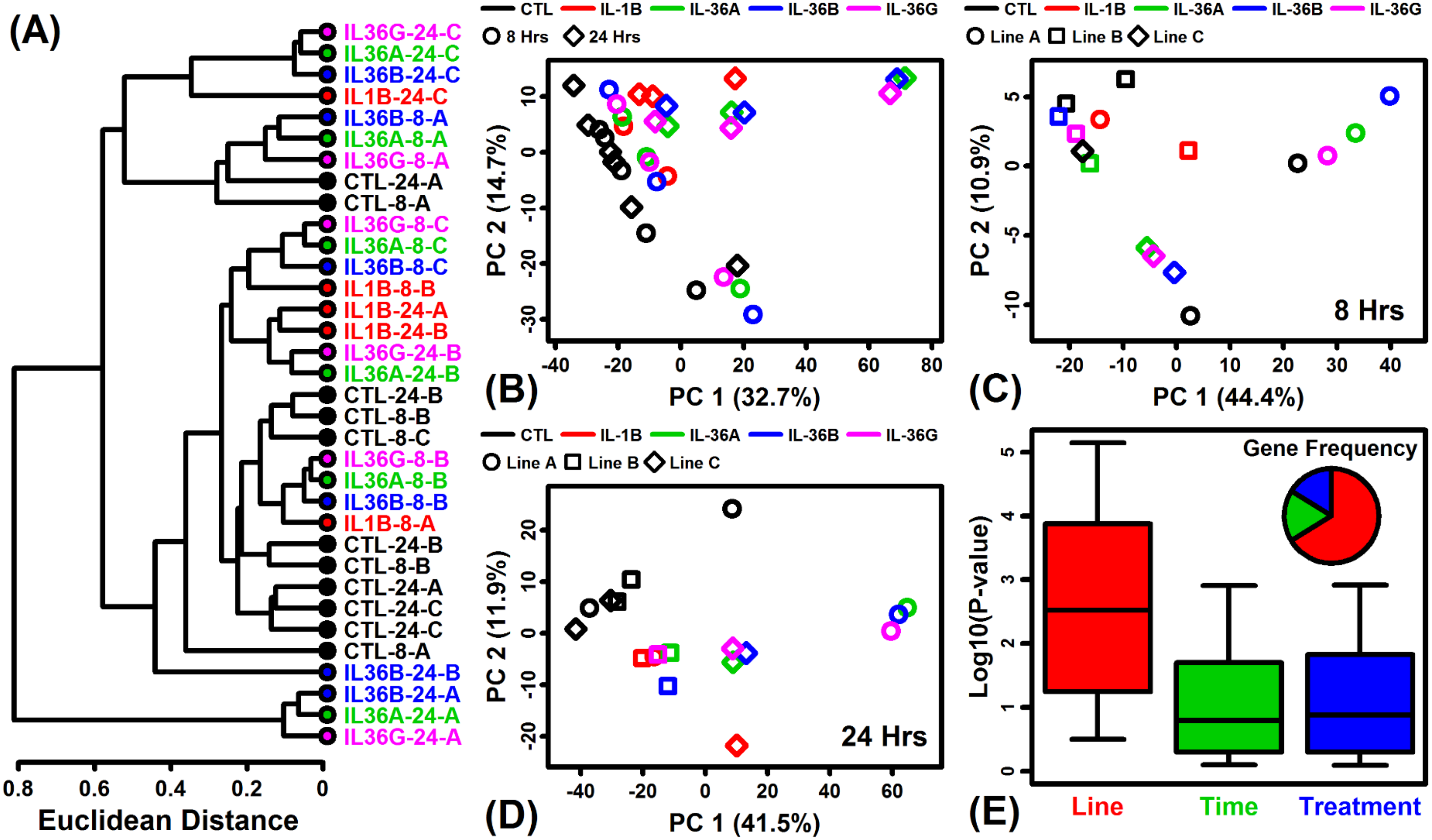

Supplemental Figure 3

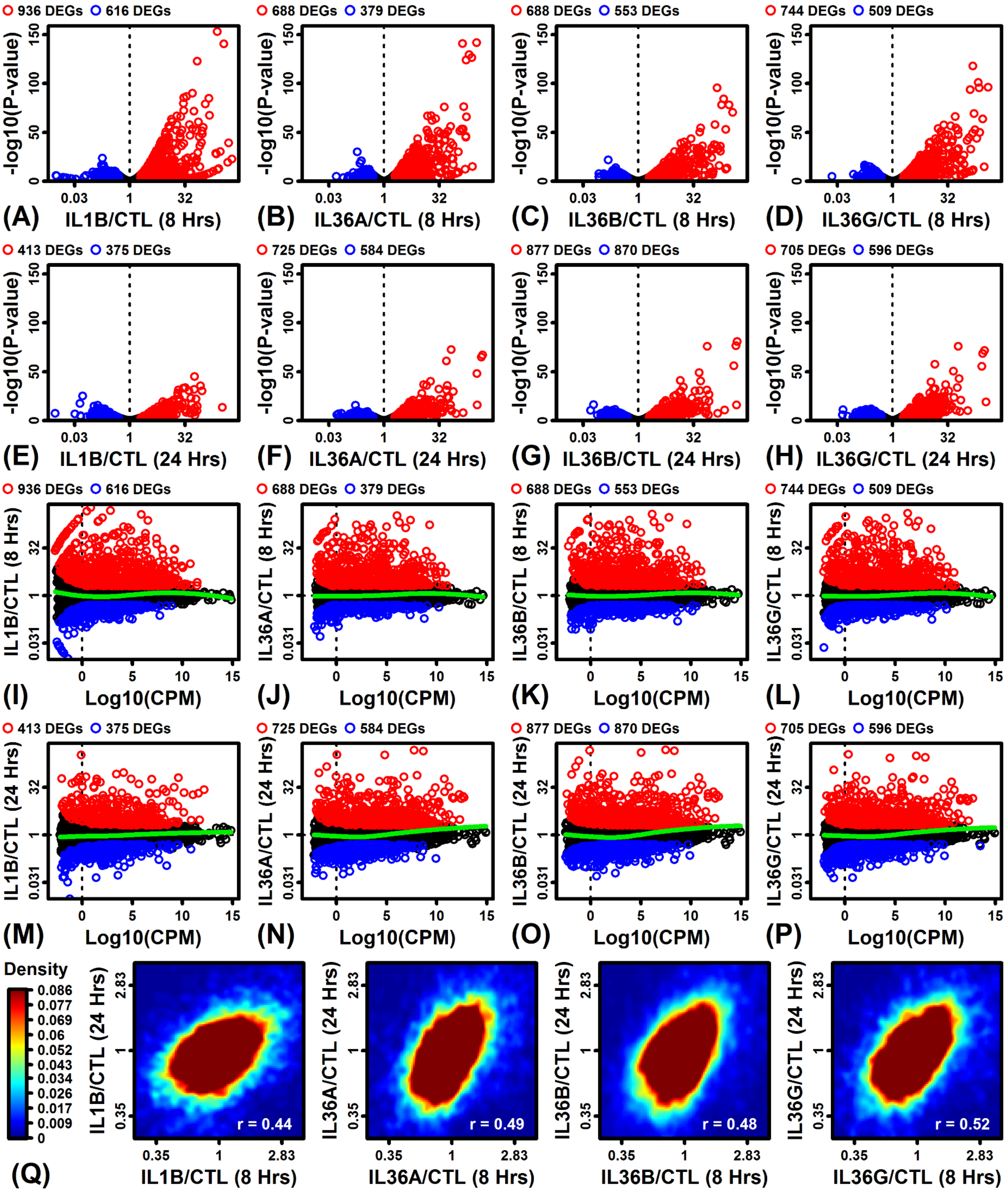

Supplemental Figure 4

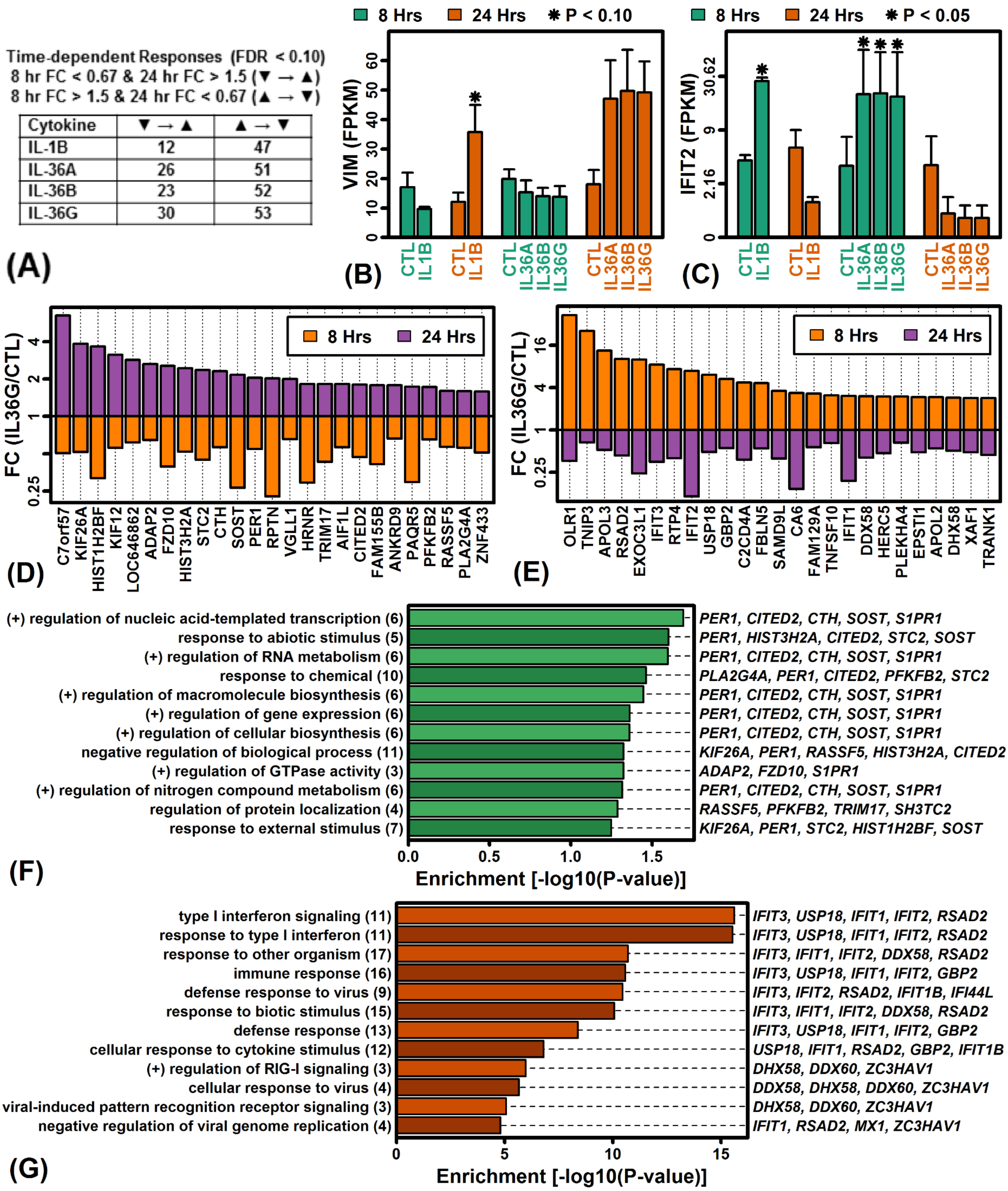

Supplemental Figure 5

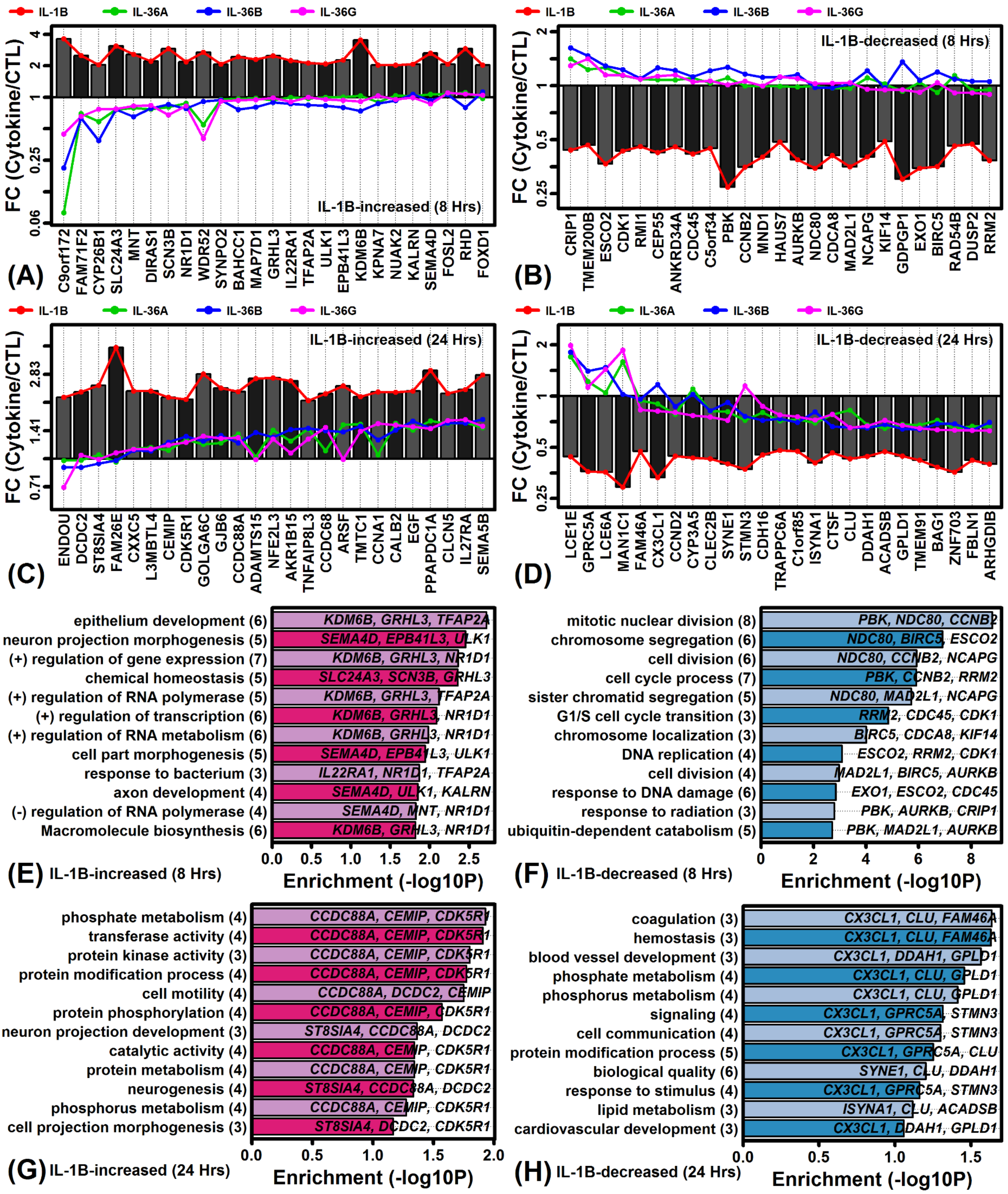

Supplemental Figure 6

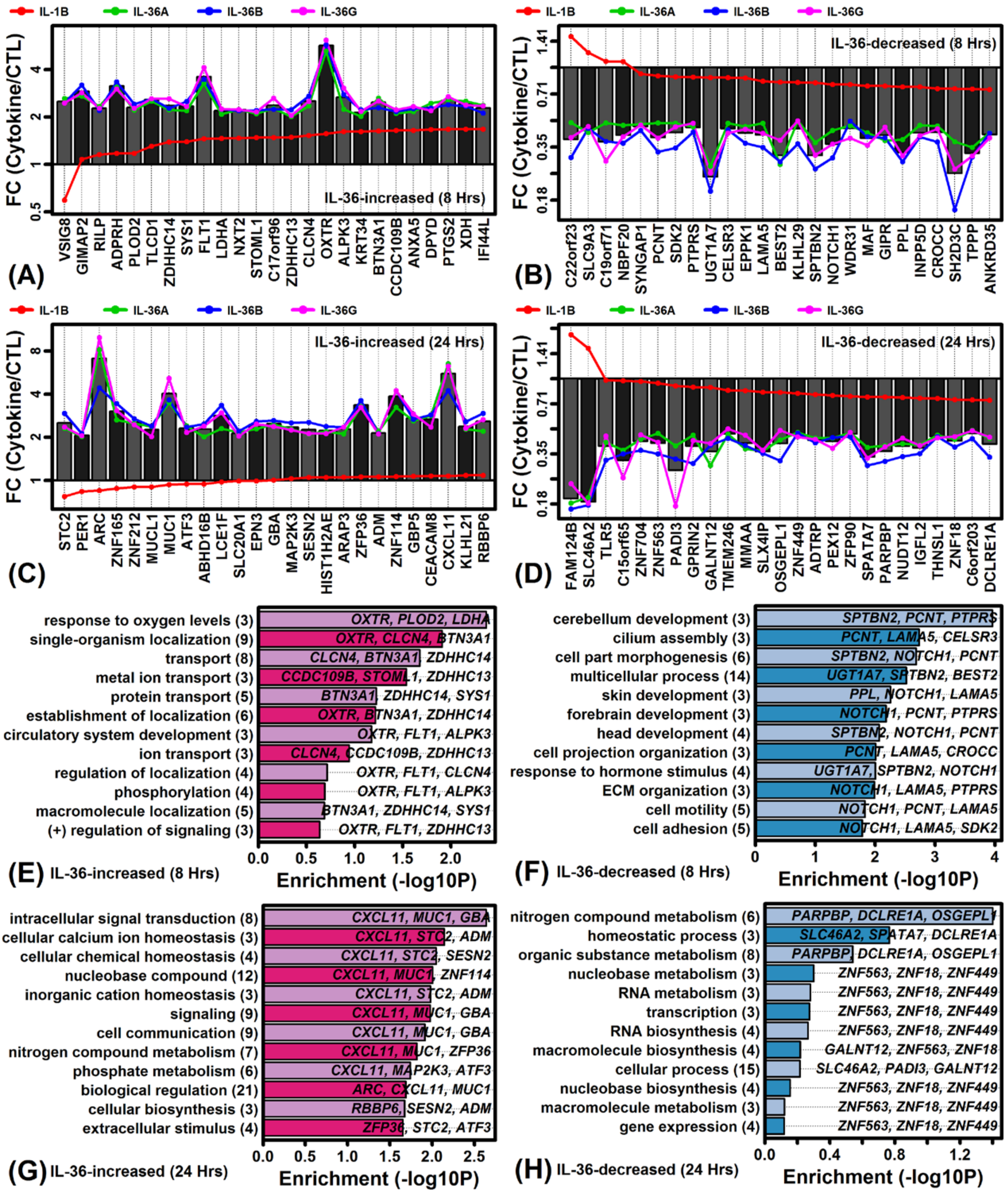

Supplemental Figure 7

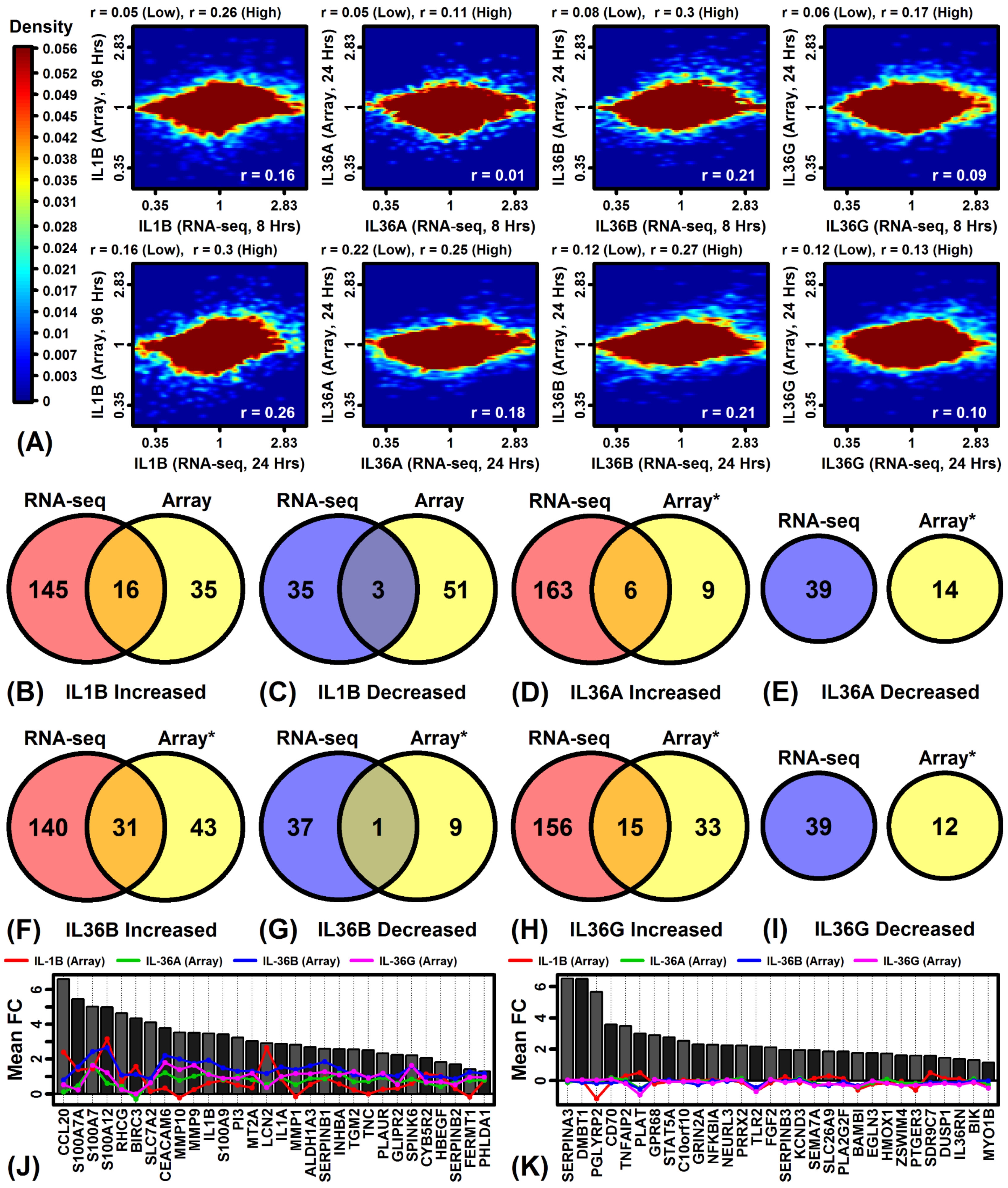

Supplemental Figure 8

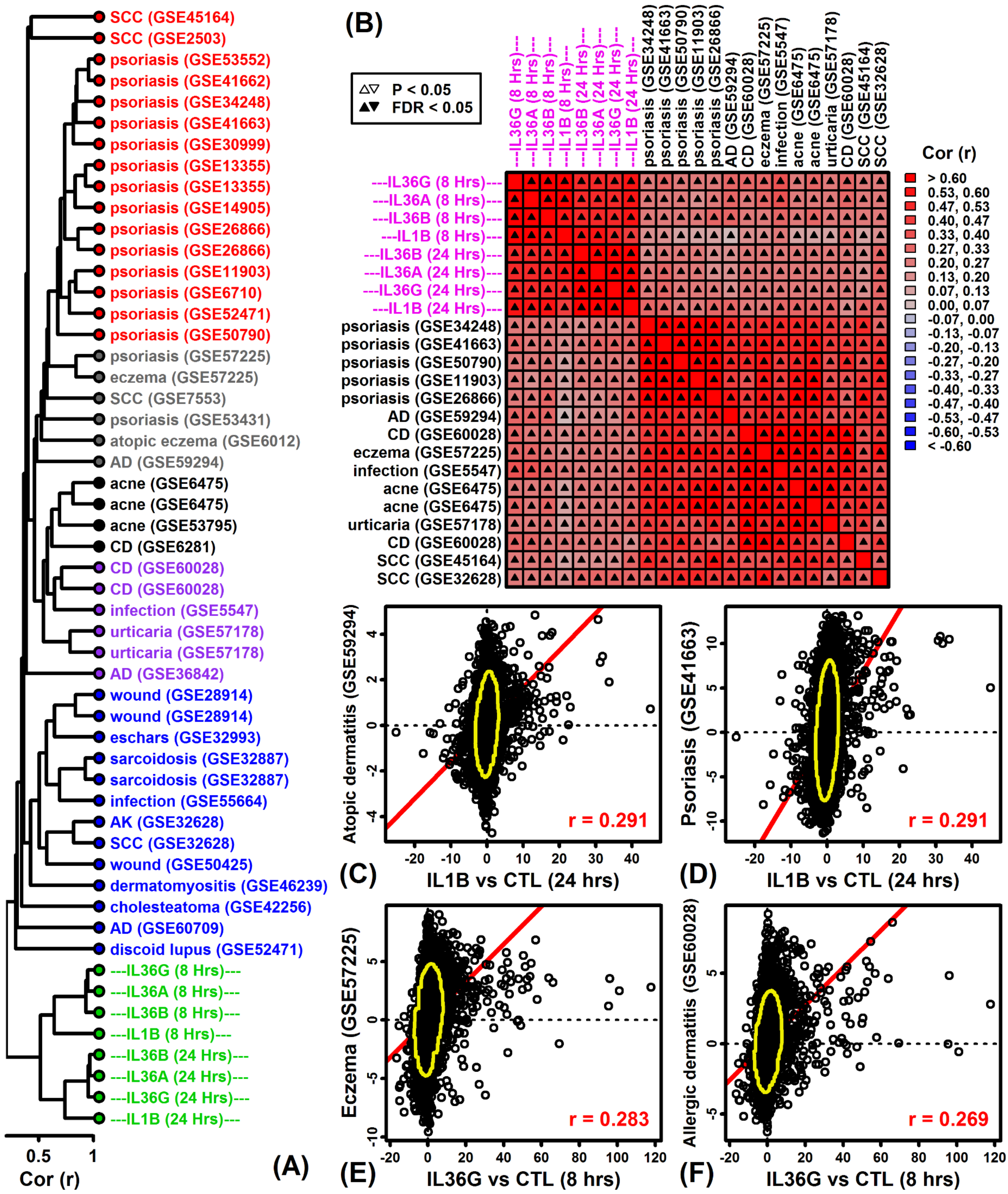

Supplemental Figure 9

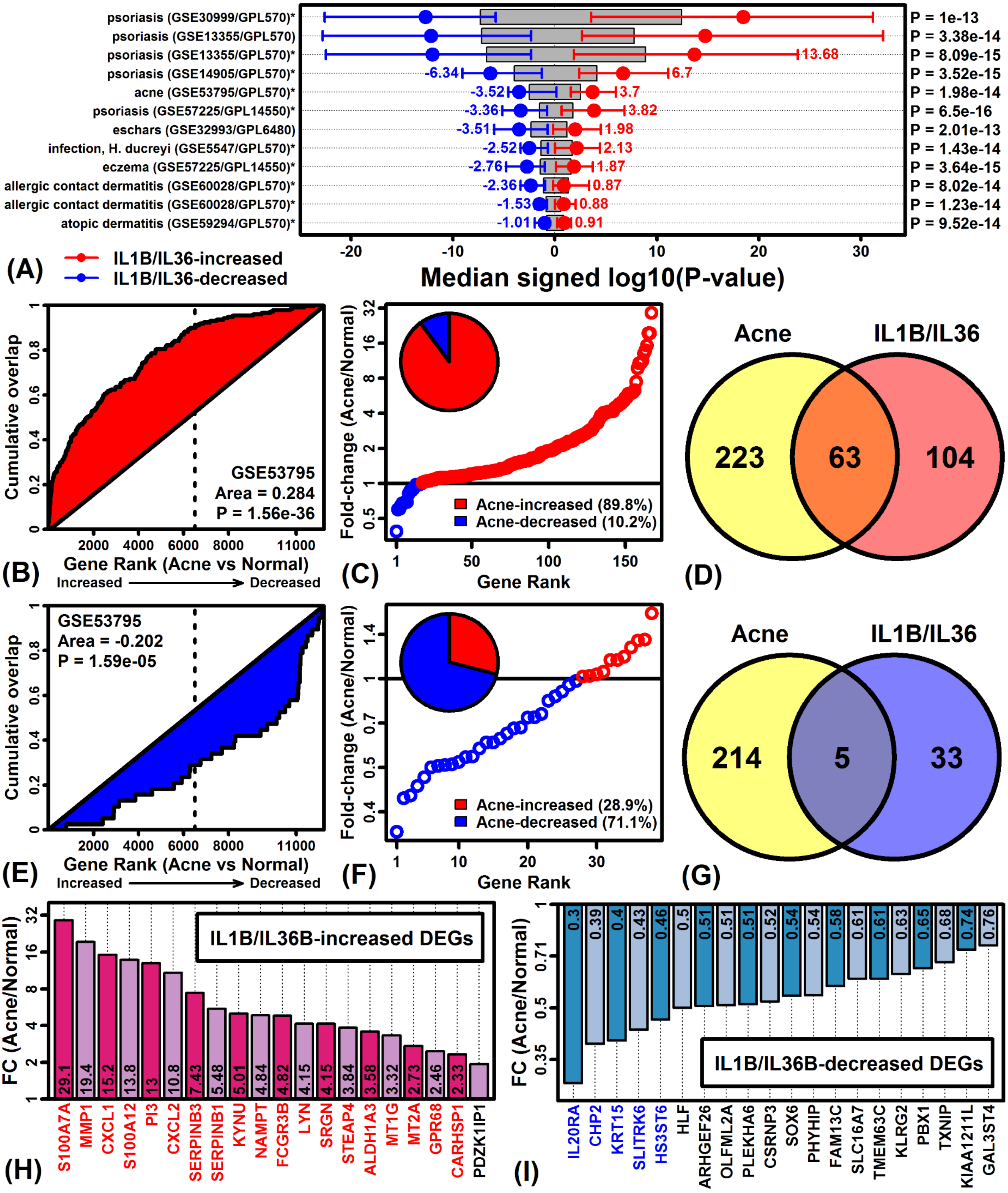

Supplemental Figure 10

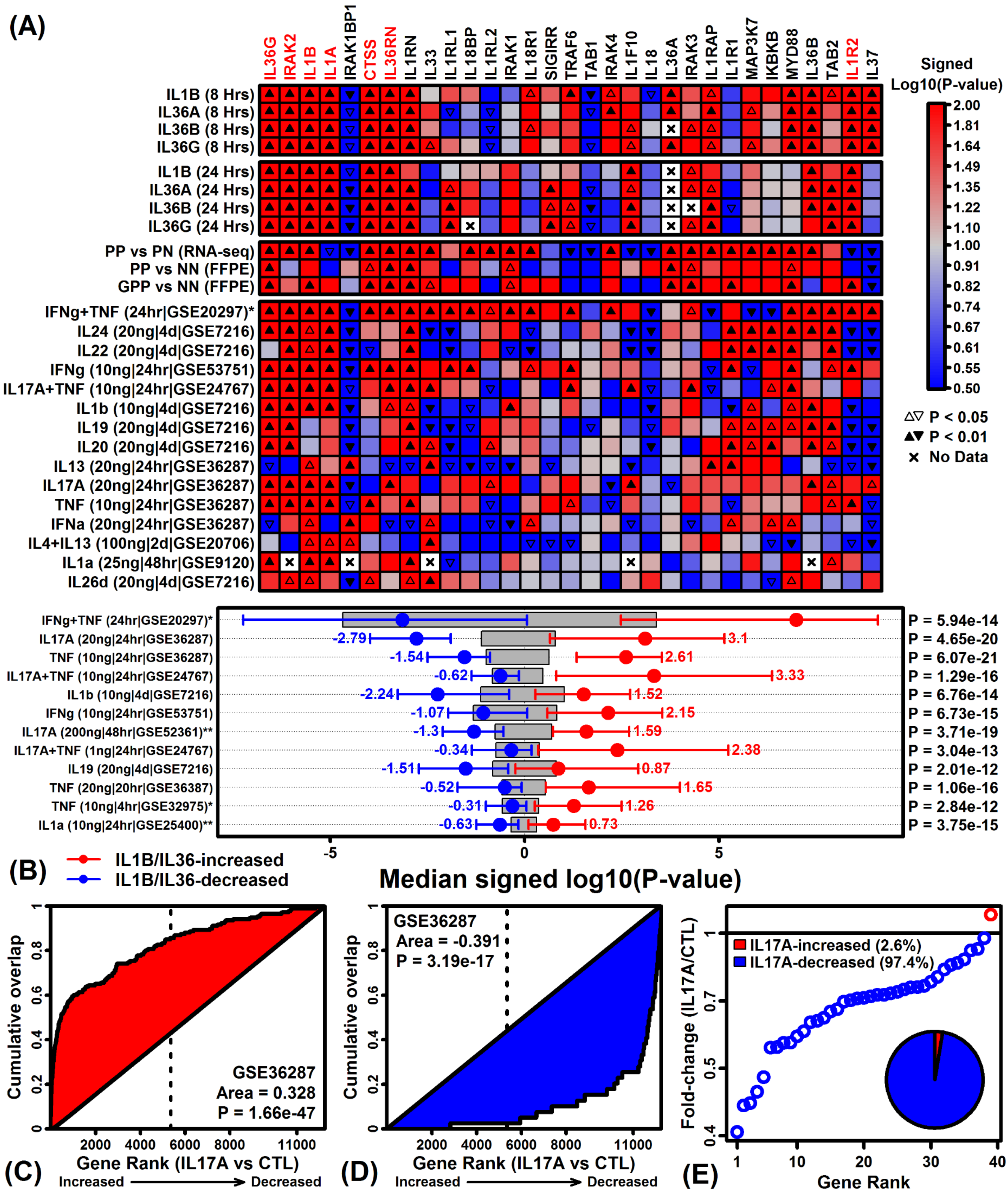

Supplemental Figure 11

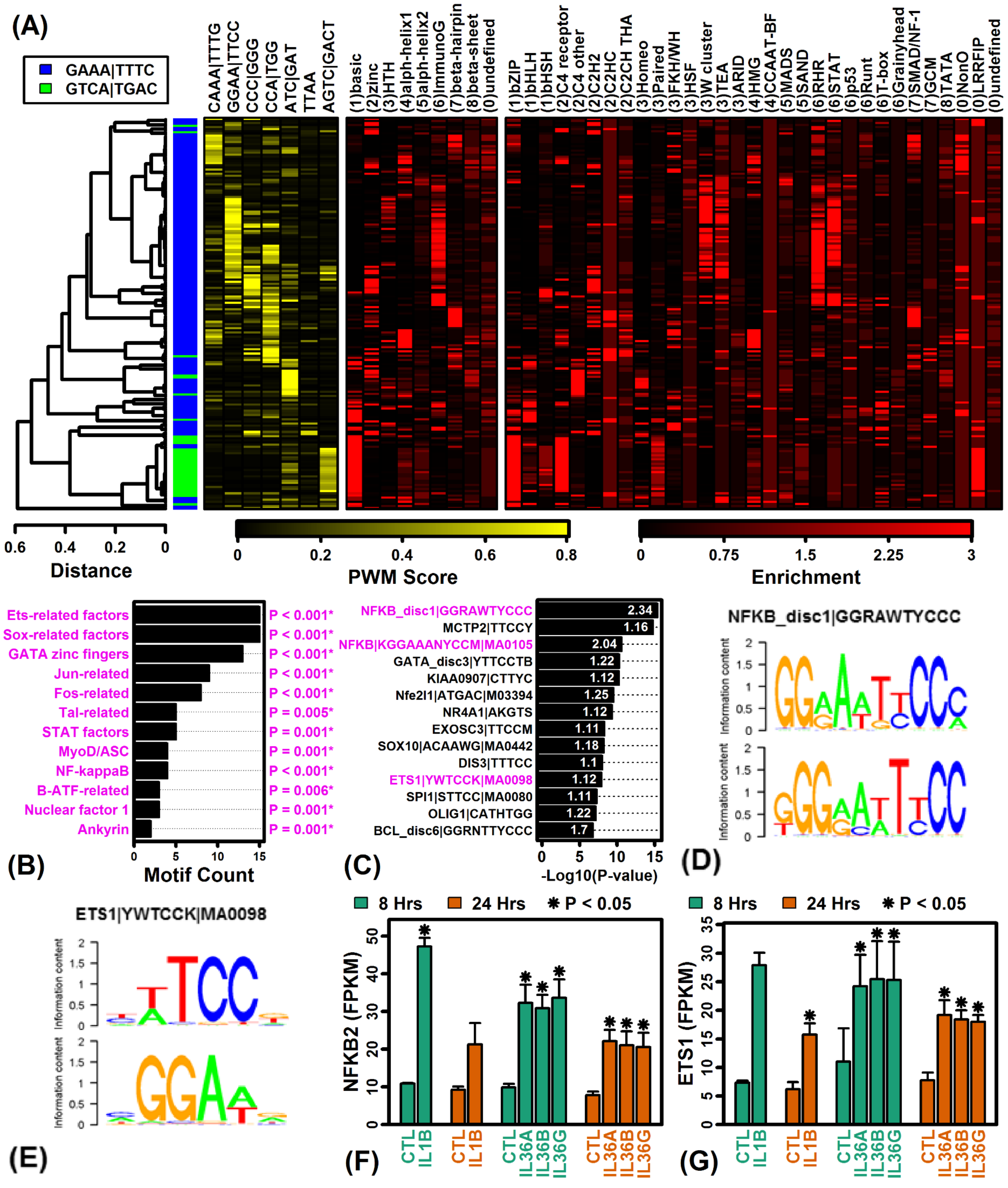

Supplemental Figure 12

(A)

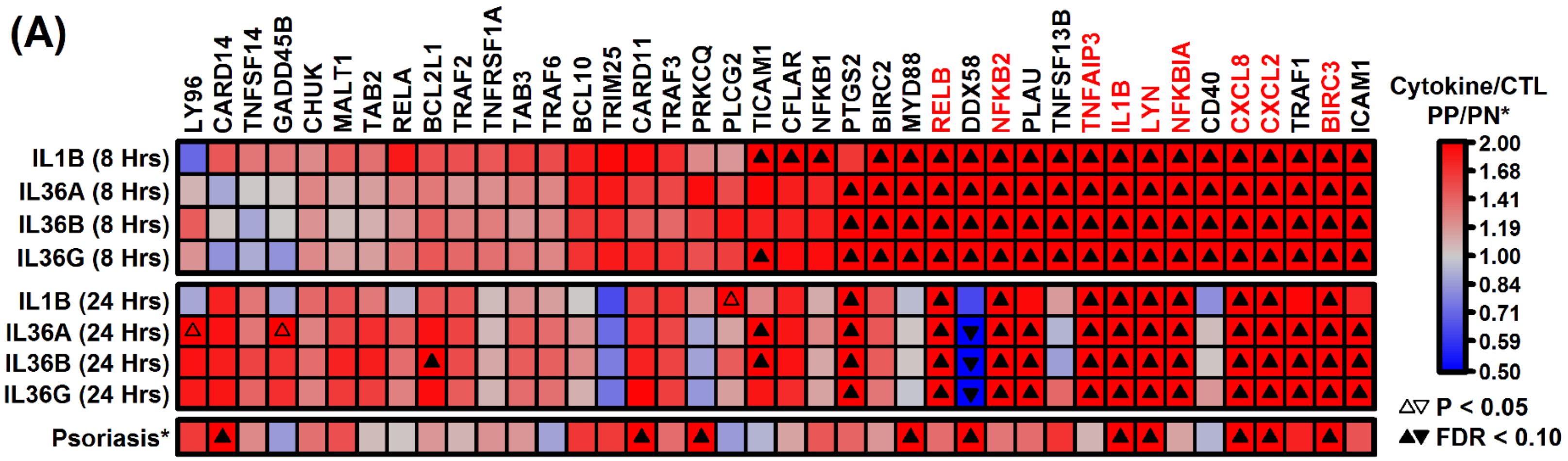

(B) NF-κB Signaling Pathway (KEGG: hsa04064)

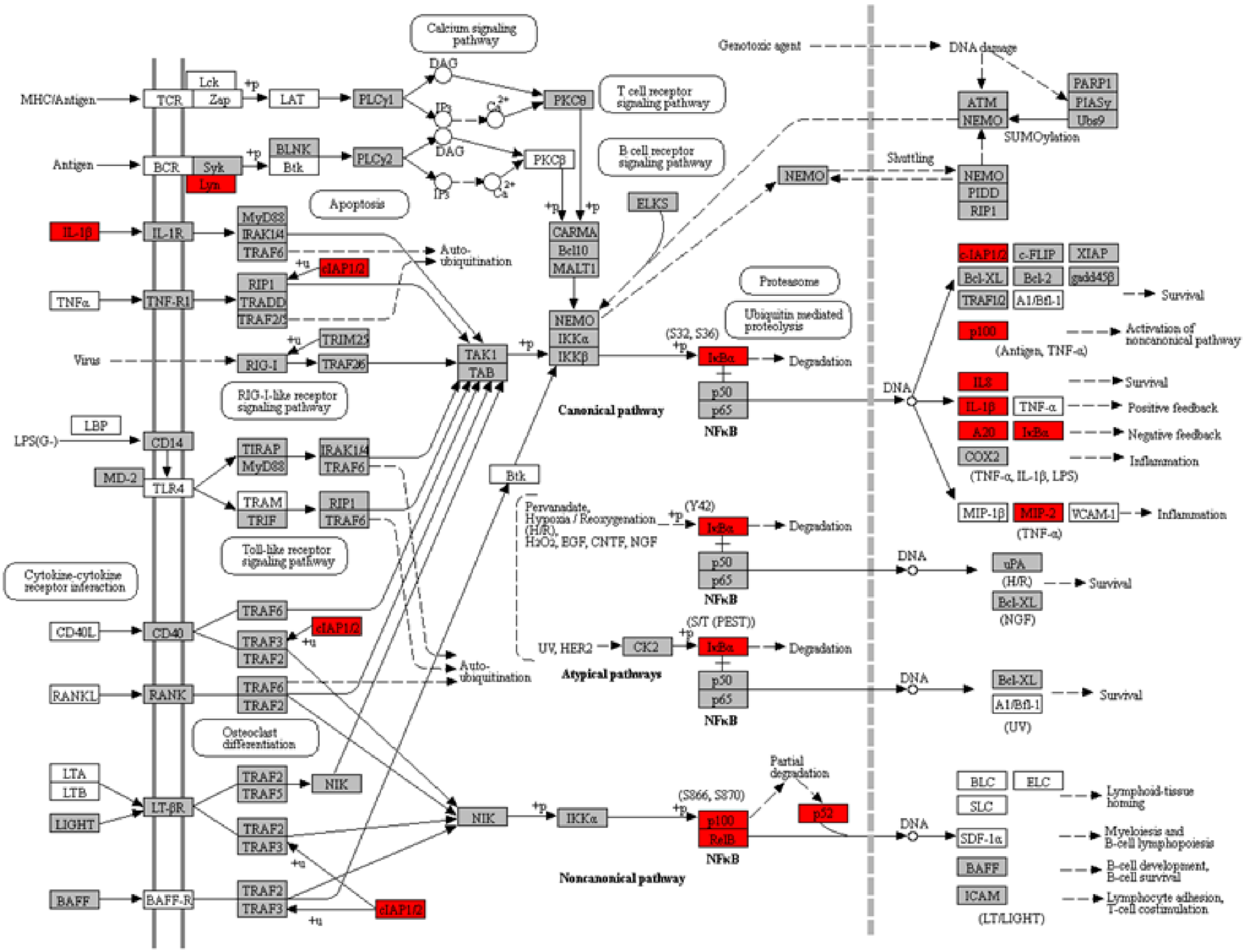

Supplemental Figure 13

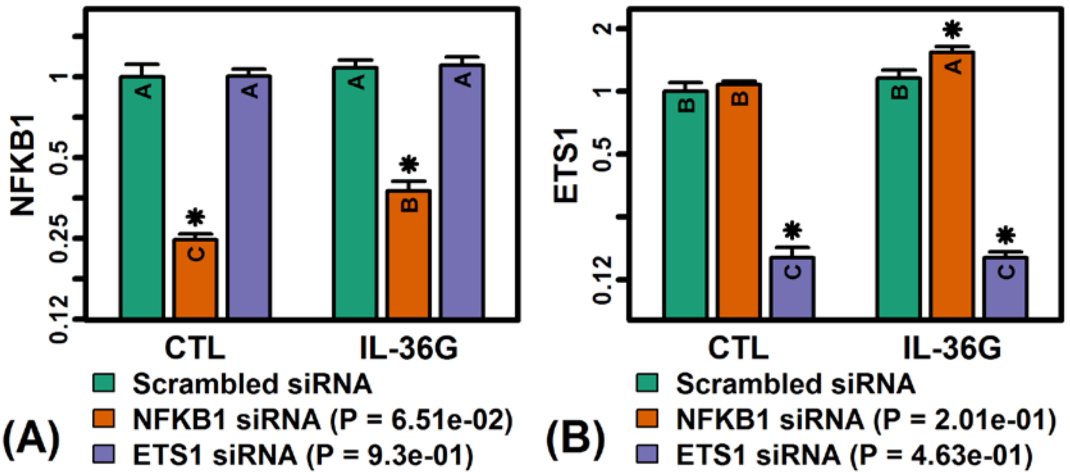

Supplemental Figure 14

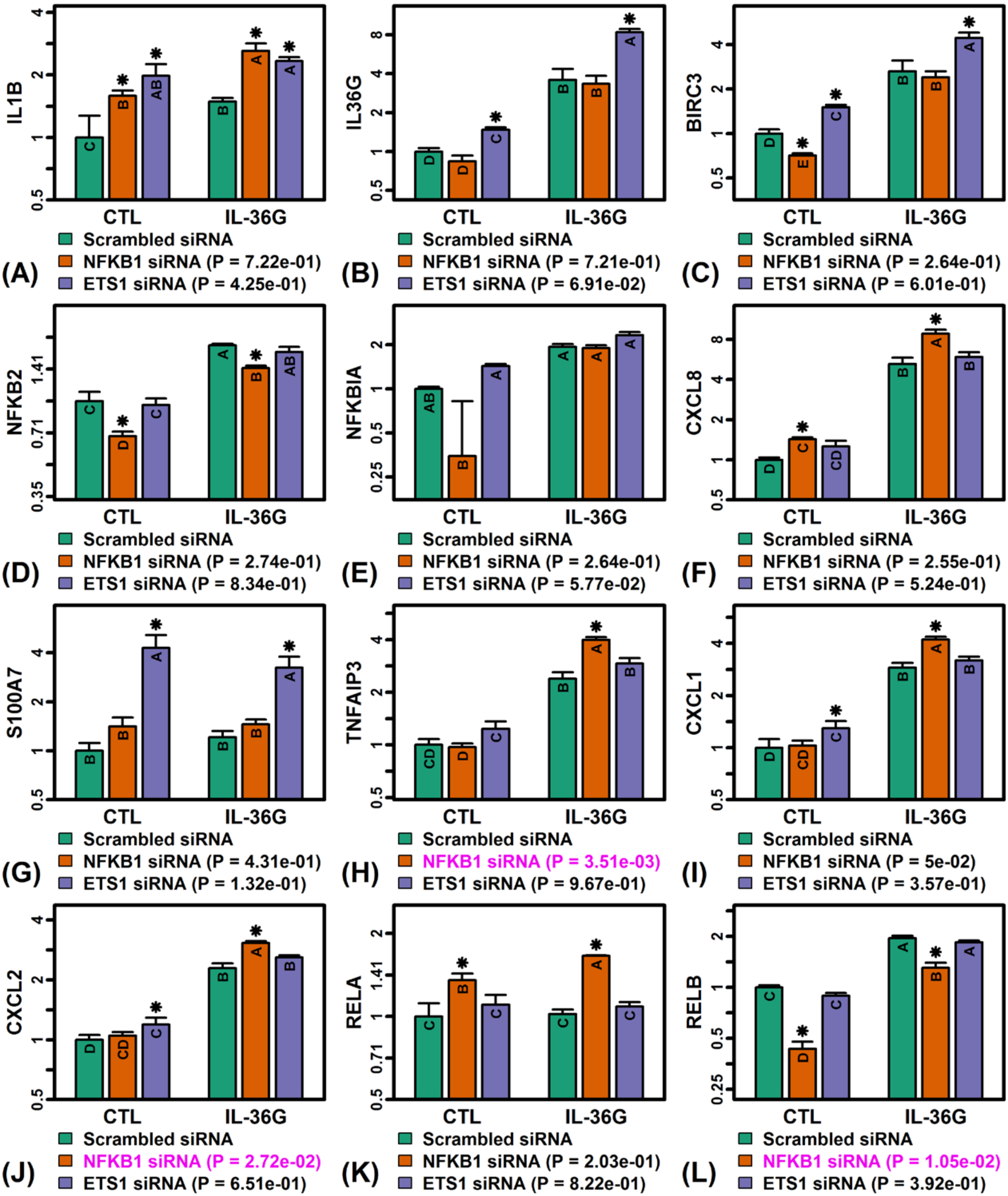

Supplement: Supplementary file 1 [file Data_Sheet_1.PDF]
